# HACE1-mediated NRF2 activation causes enhanced malignant phenotypes and decreased radiosensitivity of glioma cells

**DOI:** 10.1038/s41392-021-00793-z

**Published:** 2021-11-24

**Authors:** Chenxing Da, Jun Pu, Zhe Liu, Jing Wei, Yiping Qu, Yongxing Wu, Bingyin Shi, Jian Yang, Nongyue He, Peng Hou

**Affiliations:** 1 Key Laboratory for Tumor Precision Medicine of Shaanxi Province and Department of Endocrinology, The First Affiliated Hospital of Xi’an Jiaotong University, 710061 Xi’an, People’s Republic of China; 2 Department of Diagnostic Radiology, The First Affiliated Hospital of Xi’an Jiaotong University, 710061 Xi’an, People’s Republic of China; 3 Department of Neurosurgery, The First Affiliated Hospital of Xi’an Jiaotong University, 710061 Xi’an, People’s Republic of China; 4 State Key Laboratory of Bioelectronics, Southeast University, 210096 Nanjing, People’s Republic of China; 5 Shanxi Provincial Crops Hospital of Chinese People’s Armed Police Force, 710054 Xi’an, People’s Republic of China

**Keywords:** Cancer, Molecular biology

## Abstract

HACE1, an E3 ubiquitin-protein ligase, is frequently inactivated and has been evidenced as a putative tumor suppressor in different types of cancer. However, its role in glioma remains elusive. Here, we observed increased expression of HACE1 in gliomas related to control subjects, and found a strong correlation of high HACE1 expression with poor prognosis in patients with WHO grade III and IV as well as low-grade glioma (LGG) patients receiving radiotherapy. HACE1 knockdown obviously suppressed malignant behaviors of glioma cells, while ectopic expression of HACE1 enhanced cell growth in vitro and in vivo. Further studies revealed that HACE1 enhanced protein stability of nuclear factor erythroid 2-related factor 2 (NRF2) by competitively binding to NRF2 with another E3 ligase KEAP1. Besides, HACE1 also promoted internal ribosome entry site (IRES)-mediated mRNA translation of NRF2. These effects did not depend on its E3 ligase activity. Finally, we demonstrated that HACE1 dramatically reduced cellular ROS levels by activating NRF2, thereby decreasing the response of glioma cells to radiation. Altogether, our data demonstrate that HACE1 causes enhanced malignant phenotypes and decreased radiosensitivity of glioma cells by activating NRF2, and indicate that it may act as the role of prognostic factor and potential therapeutic target in glioma.

## Introduction

As the most common form of brain tumors, glioma has the features of infiltrative growth, hards to be resected completely, easy recurrence, and poor prognosis.^1^ Despite the progress in the treatment of glioma, the prognosis is still disappointing.^2–4^ Thus, to develop more effective therapeutic strategies, determining the potential mechanism of glioma pathogenesis becomes more pressing.

Homologous to the E6-AP carboxyl terminus (HECT) domain and Ankyrin repeat Containing E3 ubiquitin-protein ligase 1 (HACE1) is involved in cardioprotection and resistance to oxidative stress, and been proved to be frequently downregulated or mutated in different types of cancer, implying that it may function as a putative tumor suppressor.^5–8^ As supported, Hace1^−/−^ mice spontaneously develop different tumors.^4^ Further in vitro and in vivo studies have shown that Hace1 inhibits cell cycle progression of different cancer cells by decreasing the expression of cyclin D1.^6,9^

As a substrate of Hace1, Rho GTPase Rac1 can be ubiquitylated at lysine 147 by HACE1.^10^ Many studies have indicated that Rac1 is involved in regulating various biological processes, including cell motility, cell–cell contact, protein translation, and cell growth.^11,12^ A previous study evidenced that HACE1 ubiquitylated optineurin, thereby suppressing in vitro and in vivo growth of lung cancer cells by activating the autophagy.^13^ On the other hand, HACE1 also displayed pro-invasive properties in melanoma cells.^14^ However, its biological function in glioma remains to be determined.

The present study finds a strong correlation of high HACE1 expression with poor survival in patients with WHO grade III and IV. Further studies reveal that HACE1 causes enhanced malignant phenotypes and decreased radiosensitivity of glioma cells by activating nuclear factor erythroid 2-related factor 2 (NFE2L2, also known as NRF2) via multiple mechanisms.

## Results

### A strong relationship between high HACE1 expression and poor prognosis in glioma patients

**Fig. 1:**
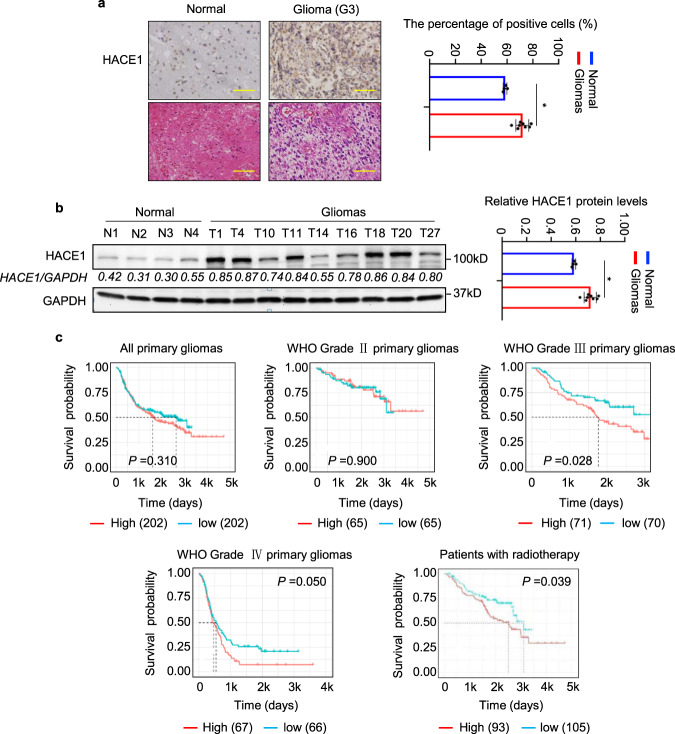
Correlation between increased expression of HACE1 and poor prognosis in LGG patients. **a** Left panel: immunohistochemistry staining (×40) showing HACE1 expression in paraffin-embedded gliomas (*n* = 9) and normal brain tissues (*n* = 4). Right panel showing quantitative illustration of HACE1 proteins. Scale bars, 200 μm. **b** Left panel: western blot analysis of HACE1 expression in fresh-frozen gliomas (*n* = 9) and normal brain tissues (*n* = 4). GAPDH as a loading control was used to normalize protein levels of HACE1 using the Tanon GIS 1D analysis software. Right panel showing statistical analysis of HACE1 proteins. **c** The correlation between HACE1 expression and poor prognosis in glioma patients by using R-Studio and X-tile (data from CGGA data set). Data were expressed as mean ± SD. *P < 0.05

We first determined HACE1 expression by immunohistochemistry and western blot assays, and found that elevated expression of HACE1 in gliomas compared to normal brain tissues (control subjects) (Fig. 1a, b). By analyzing the RNA-Seq data set from the Chinese Glioma Genome Altas (CGGA), we found that high HACE1 expression clearly reduced the survival in patients with WHO grade III and IV (Fig. 1c). Further analysis indicated a close association of high HACE1 expression with poor survival in low-grade glioma (LGG) patients receiving radiotherapy (Fig. 1c). Due to the limited number of samples, we did not investigate the correlation of HACE1 expression with the survival of high-grade glioma (HGG) patient receiving radiotherapy. These observations suggest that HACE1 may affect the response of glioma patients to radiotherapy.

### HACE1 enhances malignant behaviors of glioma cells in an E3 ligase-independent manner

**Fig. 2:**
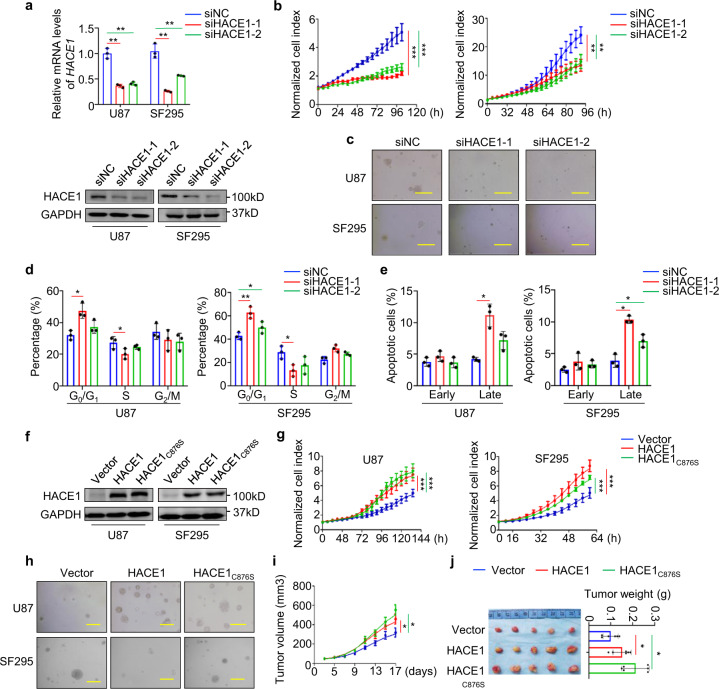
Oncogenic role of HACE1 in glioma. **a** Inhibition of HACE1 expression by two different siRNAs targeting HACE1 (si-HACE1-382 and si-HACE1-854) in SF295 and U87 cells was validated by qRT-PCR and western blot assays with 18S rRNA and GAPDH as the normalized controls. **b** The proliferation of HACE1 kncokdown-U87 and SF295 cells and control cells was monitored by the iCELLigence system. **c** Colony formation assays of HACE1 kncokdown-U87 and SF295 cells and control cells. Scale bars, 200 μm. Cell cycle (**d**) and apoptosis (**e**) assays of HACE1 kncokdown-U87 and SF295 cells and control cells by flow cytometry. **f** Western blot analysis confirms ectopic expression of HACE1 or HACE1_C876S_ in SF295 and U87 cells. GAPDH was used as a loading control. Cell proliferation (**g**) and colony formation (**h**) assays of HACE1-overexpression cells and control cells. **i** Tumor growth curves in nude mice were compared between HACE1/HACE1_C876S_-overexpression-U87 cells and control cells (*n* = 5/group). **j** Left panel: representative images of xenograft tumors; right panel: statistical results of tumor weight in HACE1/HACE1_C876S_-overexpression and control mice. Data were expressed as mean ± SD. *P < 0.05; **P < 0.01; ***P < 0.001

To determine the role of HACE1 in glioma cells, we first designed two different siRNAs targeting HACE1 (siHACE1-1 and siHACE1–2), and validated their knockdown efficiency in U87 and SF295 cells (Fig. 2a). Our data indicated that knocking down HACE1 in U87 and SF295 cells obviously suppressed cell viability and proliferation (Fig. 2b and Supplementary Fig. S1a) and cell transformation compared with the control, as reflected by anchorage-independent colony formation on soft agar (Fig. 2c, and Supplementary Fig. S1b). Meanwhile, we also observed that HACE1 knockdown caused G_0_/G_1_ cell cycle arrest (Fig. 2d) and late-stage cell apoptosis in these cells (Fig. 2e). Conversely, overexpression of HACE1 and the enzymatically inactive mutant HACE1_C876S_ in U87 and SF295 cells promoted cell proliferation and viability, and enhanced colony formation ability. (Fig. 2f–h and Supplementary Fig. S1c, d).

We also tested the effects of HACE1 and HACE1_C876S_ on tumor growth in nude mice, and found that xenograft tumors produced by HACE1/HACE1_C876S_ overexpression-U87 cells grew faster (Fig. 2i) and exhibited larger tumor weight (Fig. 2j) than control tumors. Similarly, we ectopically expressed HACE1 and HACE1_C876S_ in C6 cells, and demonstrated that ectopic expression of HACE1 or HACE1_C876S_ expectedly enhanced in vitro and in vivo cell growth (Supplementary Fig. S2). Ki-67 staining in xenograft tumors further supported the above conclusions (Supplementary Fig. S3).

Considering that high invasiveness/metastasis ability of glioma cells is a major cause for a high incidence of recurrence, meaning a worse patient survival,^15,16^ we thus investigated the influence of HACE1 on the migration and invasion of U87 and SF295 cells. Our data indicated that HACE1-knockdown cells exhibited a decreased migration/invasion potential compared to control cells (Supplementary Fig. S4a), while ectopic expression of HACE1 or HACE1_C876S_ in these cells strongly enhanced cell migration/invasion ability (Supplementary Fig. S4b). These data indicate that HACE1 enhances malignant behaviors of glioma cells in an E3 ligase-independent manner.

### HACE1 plays an oncogenic function by post-transcriptional activation of NRF2

**Fig. 3:**
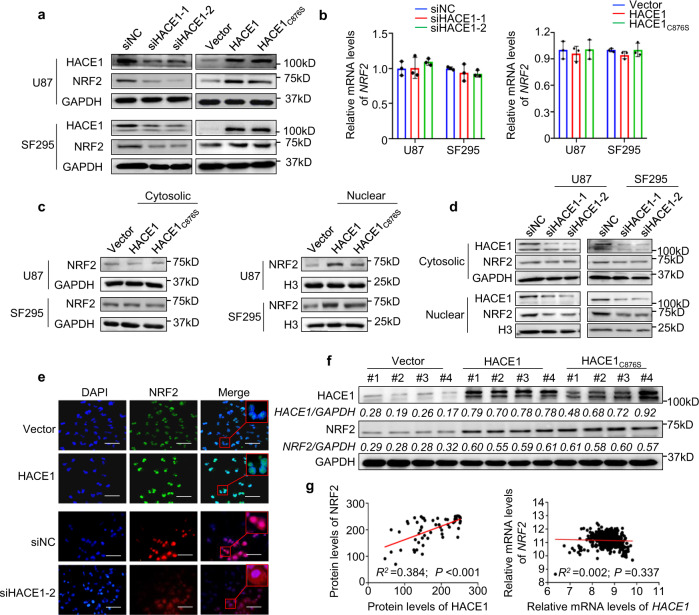
HACE1 post-transcriptionally activates NRF2. **a**, **b** NRF2 expression was analyzed in HACE1 knockdown- or HACE1/HACE1_C876S_ overexpression-SF295 and U87 cells, and control cells by western blot and qRT-PCR assays. GAPDH and 18S rRNA were used as the normalized controls, respectively. **c**, **d** Western blot analysis of NRF2 in the cytosolic and nuclear fractions of HACE1/HACE1_C876S_ overexpression- or HACE1 knockdown-SF295 and U87 cells, and control cells. GAPDH and Histone H3 were used for loading controls, respectively. **e** Immunofluorescence was carried out to determine the effect of overexpression and knockdown of HACE1 on NRF2 localization in SF295 cells. Red squares indicate the magnifications of the area. Scale bars, 20 μm. **f** Western blot analysis of HACE1 and NRF2 in xenograft tumors. The densitometry ratio of HACE1 or NRF2 to GAPDH (loading control) was shown below the corresponding band. **g** The correlation between *HACE1* and *NRF2* mRNA levels was analyzed by using the TCGA RNA-Seq data set (left panel). The relationship between protein levels of HACE1 and NRF2 was determined by western blot analysis in a panel of gliomas (right panel)

There are evidences showing that HACE1 reduces oxidative stress by activating NRF2,^17^ and NRF2 accelerates malignant transformation and progression of glioma by its anti-oxidative stress function.^18,19^ This was strongly supported by our data and the Cancer Genome Atlas (TCGA) database showing that NRF2 was significantly elevated in gliomas compared with control subjects (Supplementary Fig. S5a, b), and high NRF2 expression was strongly related to poor patient survival (Supplementary Fig. S5c). Therefore, we speculate that HACE1 enhances malignant phenotypes of glioma cells probably by regulating NRF2 activity. As shown in Fig. 3a, HACE1 depletion significantly reduced protein expression of NRF2 in U87 and SF295 cells. Conversely, ectopic expression of HACE1 or HACE1_C876S_ substantially increased protein levels of NRF2. However, knockdown or ectopic expression of HACE1 in these cells almost did not change its mRNA levels (Fig. 3b).

Further studies demonstrated that overexpression of HACE1 or HACE1_C876S_ in U87 and SF295 cells almost did not affect cytosolic protein levels of NRF2, while clearly elevated its nuclear protein levels (Fig. 3c). Conversely, knocking down HACE1 in these cells decreased nuclear protein levels of NRF2, but not its cytosolic protein levels (Fig. 3d). This was also supported by immunofluorescence assays (Fig. 3e). Besides, our results showed that overexpression of HACE1 or HACE1C876S significantly elevated mRNA expression of NRF2 targets *HMOX1* and *NQO1*, indicating the involvement of HACE1 in regulating NRF2 activity (Fig. S6). These data indicate that HACE1 promotes post-transcriptional activation of NRF2 by an E3 ligase-independent mechanism.

Consistently with the above, we demonstrated that NRF2 protein levels were clearly elevated in HACE1/HACE1_C876S_ overexpression-xenograft tumors compared to control tumors (Fig. 3f and Supplementary Fig. S7). As supported, we found a positive relationship between HACE1 and NRF2 expression at protein levels (Fig. 3g, left panel), but not at mRNA levels (Fig. 3g, right panel; data from TCGA database). These data imply that HACE1 exerts its oncogenic role probably by activating NRF2. To prove this, we knocked down NRF2 on the basis of HACE1 overexpression in U87, SF295, or U251 cells. As expected, we found that overexpression of HACE1 in glioma cells substantially enhanced the ability of cell proliferation and invasiveness compared with the control, and these effects were effectively attenuated upon NRF2 knockdown (Supplementary Fig. S8). Meanwhile, we ectopically expressed NRF2 on the basis of HACE1 knockdown in U87, SF295, or U251 cells. The results further supported the above conclusions (Supplementary Fig. S9).

### HACE1 interacts with and stabilizes NRF2 via attenuating its ubiquitin degradation

**Fig. 4:**
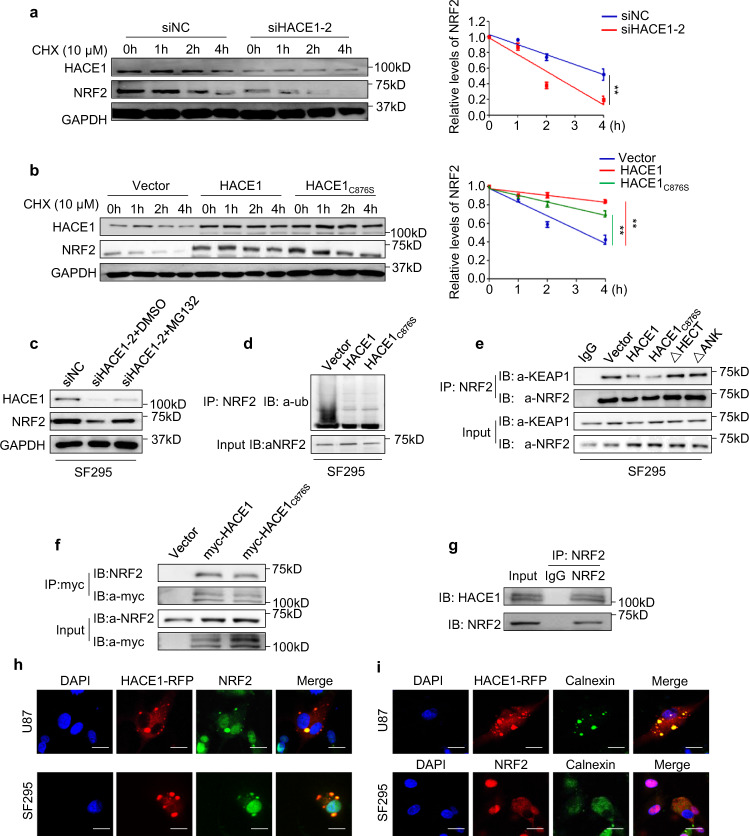
HACE1 interacts with and attenuates ubiquitin degradation of NRF2. **a**, **b** Treatment of the HACE1 knockdown- or HACE1/HACE1_C876S_ overexpression-SF295 cells and control cells with 10 μM cycloheximide at the time indicated. Left panels represent western blot analysis of the indicated proteins. The band intensity of NRF2 in cycloheximide-treated cells was normalized to that of GAPDH (loading control), and subsequently normalized to that in DMSO-treated cells (right panels). Data were expressed as mean ± SD. *P < 0.05; **P < 0.01. **c** HACE1 knockdown-SF295 cells and control cells were pretreated with 25 µM MG132 or DMSO for 4 h, and western blot analysis was then carried out to assess protein expression of HACE1, NRF2, and GAPDH (loading control). **d** HACE1/HACE1_C876S_ overexpression-SF295 cells and control cells were pretreated with 25 µM MG132 for 4 h. Next, cells were lysated and mixed with anti-NRF2 antibody conjugated with agarose. Immunoblotting was then carried out to assess NRF2 ubiquitination using anti-ubiquitin (Ub) antibody. Input samples were taken prior to the above steps. **e** SF295 cells stably expressing wild-type HACE1 or the indicated mutants and control cells were lysated and immunoprecipitated (IP) with anti-NRF2 antibody, and the precipitated proteins were subjected to western blot analysis using anti-KEAP1 antibody. **f**, **g** The interaction between HACE1 and NRF2 was evaluated by reciprocal Co-IP assays in HACE1/HACE1_C876S_ overexpression-SF295 cells and control cells. **h** Immunofluorescence staining showing the co-localization of HACE1 and NRF2 in the extranuclear region of SF295 and U87 cells. Blue color represents DAPI staining for nuclei; Red color represents HACE1 tagged with red fluorescence protein (RFP); green color represents NRF2. **i** Immunofluorescence assay showing the co-localization of HACE1 and NRF2 in the endoplasmic reticulum of SF295 and U87 cells. Blue color represents DAPI staining for nuclei; Red color represents HACE1 tagged with RFP or NRF2; Green color represents calnexin. Scale bars, 20 μm

The above results demonstrated that HACE1 could post-transcriptionally activate NRF2. To reveal the exact mechanism, we blocked new protein synthesis by treating glioma cells with cycloheximide (CHX) to evaluate the influence of HACE1 on NRF2 protein stability. Our data showed that, relative to the control, HACE1 knockdown markedly accelerated the degradation of NRF2 proteins in SF295 cells (Fig. 4a), while ectopic expression of HACE1 or HACE1_C876S_ enhanced NRF2 protein stability (Fig. 4b). Next, proteasome inhibitor MG132 was used to block the ubiquitin-proteasome pathway in these cells. The results indicated that MG132 obviously reversed the inhibitory effect of HACE1 knockdown on NRF2 expression (Fig. 4c). Accordingly, we found that overexpression of HACE1 or HACE1_C876S_ reduced the ubiquitination of NRF2 proteins (Fig. 4d). These findings imply that HACE1 regulates NRF2 proteolysis through the ubiquitin-proteasome pathway.

It is clear that NRF2 can be degraded by the proteasome pathway via binding to KEAP1, while the latter can form an E3 ubiquitin ligase complex with Cul3.^20^ Thus, we suppose that HACE1 may impair NRF2-KEAP1 interaction and hence, stabilizes NRF2. As shown in Fig. 4e, we performed the Co-IP assays and found that overexpression of HACE1 or HACE1_C876S_ reduced the interaction between NRF2 and KEAP1 in SF295 cells, while the deletion of HECT or ANK domain in HACE1 could eliminate this effect. Based on the above results, we speculate that HACE1 may interact with NRF2 to pose a steric hindrance for the interaction between NRF2 and KEAP1. Expectedly, our data demonstrated the interaction between HACE1 and NRF2 by a reciprocal Co-IP assay (Fig. 4f, g). Besides, we found that HACE1 and NRF2 did co-localize mostly in the extranuclear region of SF295 and U87 cells, and demonstrated their interaction on the endoplasmic reticulum by immunofluorescence assays (Fig. 4h, i and Supplementary Fig. S10a). To further confirm the above conclusion, we knocked down KEAP1 in SF295 cells, and found that KEAP1 knockdown expectedly enhanced the interaction between HACE1 and NRF2 (Supplementary Fig. S10b, c). These data imply that HACE1 interacts with and stabilizes NRF2 by attenuating its ubiquitin degradation.

### HACE1 enhances internal ribosome entry site (IRES)-mediated mRNA translation of NRF2 via La/SSB

**Fig. 5:**
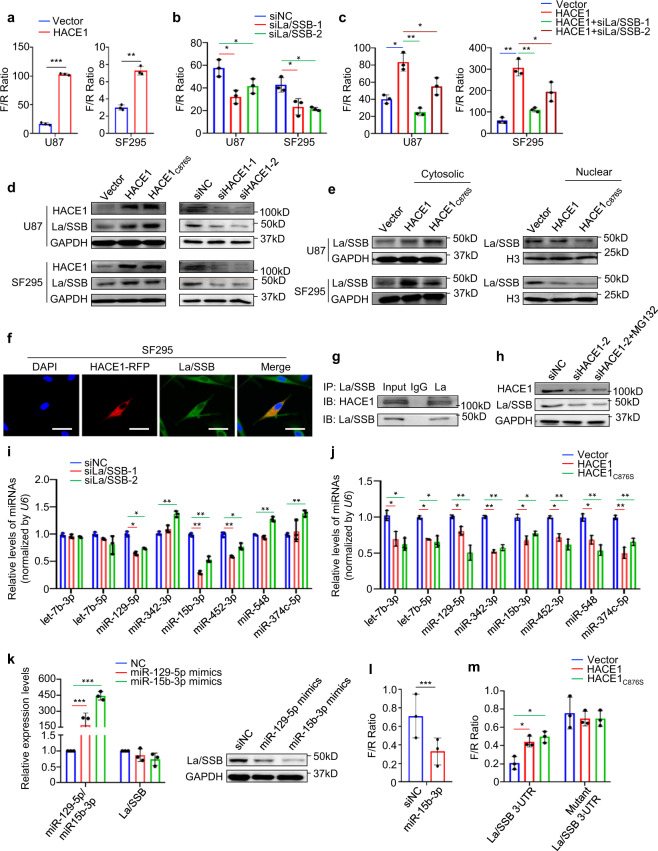
HACE1 enhances IRES-mediated translation of NRF2. **a**, **b** The IRES activity of NRF2 was detected by the dual-luciferase reporter system in HACE1 overexpression- or La/SSB knockdown-SF295 and U87 cells and control cells. **c** La/SSB was knocked down on the basis of ectopic expression of HACE1. The IRES activity of NRF2 was then assessed by the dual-luciferase reporter system. **d** Western blot analysis of HACE1, La/SSB, and GAPDH (loading control) in HACE1/HACE1_C876S_ overexpression- or HACE1 knockdown-SF295 and U87 cells, and control cells. **e** Western blot analysis of La/SSB, GAPDH (loading control for cytosolic proteins), and histone H3 (loading control for nuclear proteins) in cytosolic and nuclear fractions of HACE1/HACE1_C876S_ overexpression-U87 and SF295 cells and control cells. **f** Immunofluorescence assay showing the co-localization of HACE1 and La/SSB in the extranuclear region of SF295 cells. Blue color represents DAPI staining for nuclei; red color represents HACE1 tagged with RFP; Green color represents La/SSB. Scale bars, 20 μm. **g** Co-IP assay showing the interaction of HACE1 and La/SSB in SF295 cells. **h** HACE1 knockdown-SF295 cells and control cells were pretreated with 25 µM MG132 or DMSO for 4 h, and then subjected to western blot analysis using the indicated antibodies. **i, j** qRT-PCR assay of the indicated miRNAs in the La/SSB knockdown-, or HACE1/HACE1_C876S_ overexpression-SF295 cells and control cells. **k** The impacts of miR-129-5p and miR-15b mimics on La/SSB expression at mRNA or protein levels were assessed by qRT-PCR and western blot assays in SF295 cells. U6 and GAPDH were used as the normalized controls, respectively. **l** The effect of miR-15b mimic on *La/SSB* 3′-UTR activity was evaluated by the dual-luciferase reporter system. **m** Wild-type or mutant *La/SSB* 3′-UTR activity was evaluated by the dual-luciferase reporter system in HACE1/HACE1_C876S_ overexpression-SF295 cells and control cells. Data were expressed as mean ± SD. *P < 0.05; **P < 0.01; ***P < 0.001

There is evidence indicating that HACE1 accelerates NRF2 protein synthesis under oxidative stress.^13^ However, the mechanism is totally unclear. Increasing evidences have shown that internal ribosomal entry sites (IRESs) are structural RNA motifs that initiate translation in the middle of a mRNA in a cap-independent manner.^21^ Coincidently, an IRES element was identified within the 5′-UTR of human *NRF2* mRNA.^21,22^ Thus, we speculate that HACE1 promotes NRF2 protein synthesis via IRES-mediated translation. To prove this, we cloned 5′-UTR of *NRF2* containing an IRES element into a dicistronic reporter construct, which was widely used to assess the IRES-mediated translation. The results showed that overexpression of HACE1 or HACE1_C876S_ substantially elevated 5′-UTR activity of *NRF2* compared with the control in U87 and SF295 cells (Fig. 5a).

A previous study showed that La/SSB initiates de novo translation of NRF2 protein by binding to the 5′-UTR of its mRNA.^23^ This was in accord with our data that La/SSB knockdown reduced *NRF2* 5′-UTR activity (Fig. 5b) and reversed the positive regulatory effect of HACE1 overexpression on *NRF2* 5′-UTR activity (Fig. 5Ic). Meanwhile, we ectopically expressed La/SSB in HACE1 knockdown-U87 and SF295 cells, and expectedly found that La/SSB overexpression reversed the inhibitory effect of HACE1 knockdown on *NRF2* 5′-UTR activity (Supplementary Fig. S11), supporting that HACE1 promotes IRES-mediated translation of NRF2 via La/SSB. Further studies showed that overexpression of HACE1 or HACE1_C876S_ increased La/SSB expression in U87 and SF295 cells, while HACE1 knockdown decreased La/SSB expression (Fig. 5d). Moreover, we also found that HACE1 or HACE1_C876S_ overexpression increased cytosolic protein levels of La/SSB, while decreased its nuclear protein levels (Fig. 5e).

The above results demonstrated that HACE1 upregulated La/SSB expression and impaired La/SSB distribution between nucleus and cytoplasm. To illustrate the corresponding mechanism, we performed immunofluorescence and Co-IP assays, and found that HACE1 interacted and co-localized mostly with La/SSB in the extranuclear region (Fig. 5f, g). In addition, our data indicated that knockdown or overexpression of HACE1 did not change mRNA levels of *La/SSB* (Supplementary Fig. S12a, b), and proteasome inhibitor MG132 could not affect the regulatory effect of HACE1 on La/SSB expression (Fig. 5h). These data indicate that HACE1 regulates La/SSB expression at post-transcriptional levels, but does not affect its protein stability. Next, we cloned *La/SSB* 3′-UTR into a dicistronic reporter construct, and found HACE1 or HACE1_C876S_ overexpression significantly enhanced *La/SSB* 3′-UTR activity in SF295 cells (Supplementary Fig. S12c).

It is well-known that La/SSB acts as a pre-micoRNA (pre-miRNA) binding protein to stabilize pre-miRNAs from nuclease-mediated decay by stem-loop recognition, thereby promoting miRNA biogenesis.^21^ We thus expected to probe the regulatory role of La/SSB on the expression of several miRNAs, which have been demonstrated to target *La/SSB* 3′-UTR.^24^ The results showed that La/SSB knockdown significantly downregulated miR-129-5p, miR-15b-3p, and miR-452-3p relative to the control in SF295 cells (Fig. 5i). Meanwhile, we found that these three miRNAs were also downregulated when HACE1 or HACE1_C876S_ were ectopically expressed in SF295 cells (Fig. 5j). Moreover, we predicted La/SSB as a potential target of miR-129-5p and miR-15b-3p using three online databases. To validate this, we transfected SF295 cells with miR-129-5p and miR-15b mimics, and found that miR-129-5p or miR-15b mimics almost did not affect mRNA levels of *La/SSB*, while significantly decreased its protein expression, particularly the latter (Fig. 5k). Next, we demonstrated that miR-15b mimics inhibited *La/SSB* 3′-UTR activity, and found that ectopic expression of HACE1 or HACE1_C876S_ enhanced wild-type *La/SSB* 3′-UTR activity, while did not change mutant *La/SSB* 3′-UTR activity (Fig. 5l, m).

The above findings indicate that HACE1 interacts with La/SSB, and impairs its distribution between nucleus and cytoplasm, decreasing its nuclear protein levels. As a result, some miRNAs targeting La/SSB such as miR-129-5p and miR-15b were downregulated, thereby increasing La/SSB expression at post-transcriptional levels. These will ultimately enhance the IRES-mediated mRNA translation of NRF2.

### HACE1 decreases the radiosensitivity of glioma cells via NRF2 activation

**Fig. 6:**
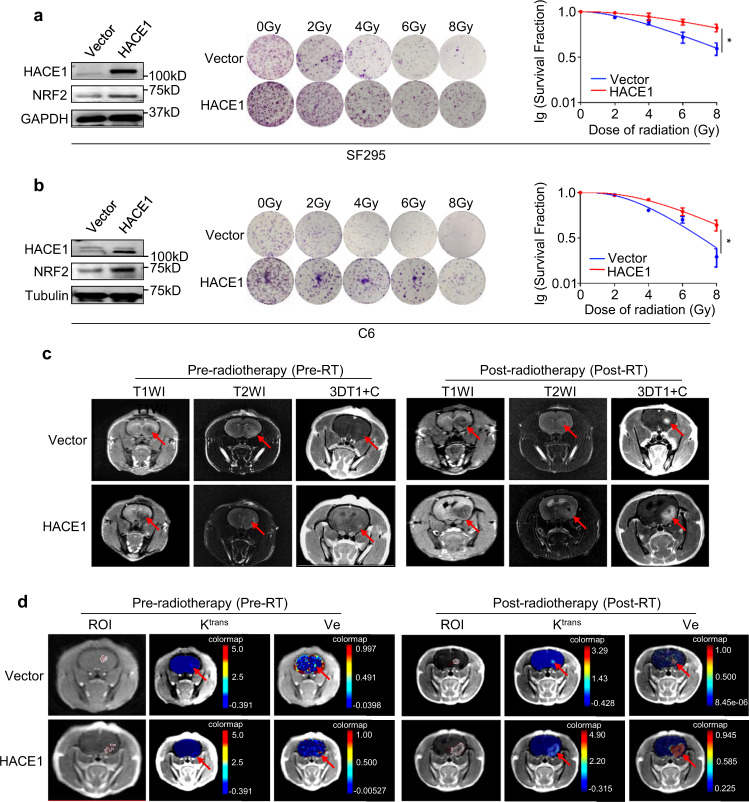
HACE1 reduces the radiosensitivity of glioma cells. **a**, **b** Western blot analysis of HACE1, NRF2, and GAPDH (loading control) in HACE1 overexpression-SF295 and C6 cells (left panel). The effect of ectopic expression of HACE1 on colony formation of SF295 or C6 cells treated with different doses of radiation (middle panel). Dose-survival curves of SF295 or C6 cells were established according to the data of colony formation assays (right panel). **c** MRI T1-weighted images, T2-weighted images, and contrast-enhanced contrast-enhanced 3-dimensional T1-weighted images in orthotopic xenograft tumors stably expressing HACE1 and control tumors showing a difference in the tumor texture. **d** DCE-MRI measures were performed in HACE1 overexpression-xenograft tumors and control tumors to evaluate radiation effect. Data were shown as mean ± SD. *P < 0.05

To determine whether HACE1 affects the response of glioma cells to radiation, we ectopically expressed HACE1 in SF295 and C6 cells, and treated these cells with radiation. The results showed that ectopic expression of HACE1 caused a lower radiosensitivity of tumor cells than the control, as reflected by cell survival (Fig. 6a, b and Supplementary Tables S1, S2).

Next, we established an orthotopic rat glioma model by injecting HACE1 overexpression-C6 cells and control cells. This model was successfully validated by H&E staining and IHC stained for HACE1, NRF2, and Ki-67 (Supplementary Fig. S13). After 12 days post-implantation, the whole brain of glioma-bearing rats were exposed to 2 Gy fractionated dose/3 times on alternate days. Before and after radiation, all tumors were investigated by MRI on T1-weighted images, T2-weighted images, and contrast-enhanced 3-dimensional T1-weighted images (Fig. 6c). The results showed that average tumor volumes in the control group increased from 2 to 22 mm^3^, while increased from 2 to 125 mm^3^ in the HACE1-overexpression group between pre-radiotherapy and post-radiotherapy (Supplementary Fig. S14). Dynamic Contrast-Enhanced MRI (DCE-MRI) were further performed to evaluate radiation effects, such as the permeability of the vessels and the vascular leakage space (Fig. 6d). Compared to pre-radiotherapy, we observed a significant increase in some parameters such as *K*^trans^ and Ve in the HACE1-overexpression group, but not in the control group after radiotherapy (Table 1). *K*^trans^ has been demonstrated to be a better predictor for post-radiation therapy response.^25,26^ These results further support that HACE1 decreases the radiosensitivity of glioma cells.

**Table 1:** Parameters using a 3D gradient-recalled echo sequence on a 3T MR scanner

	*K*^trans^-max Pre-RT	*K*^trans^-max Post-RT	*P*	*K*^trans^-mean Pre-RT	*K*^trans^-mean Post-RT	*P*	Ve-max Pre-RT	Ve-max Post-RT	*P*	Ve-mean Pre-RT	Ve-mean Post-RT	*P*
Vector	0.110 ± 0.040	0.485 ± 0.230	0.448	0.030 ± 0.010	0.267 ± 0.124	0.365	0.452 ± 0.329	0.752 ± 0.133	0.346	0.171 ± 0.129	0.4510 ± 0.178	0.454
HACE1	0.062 ± 0.033	1.322 ± 0.275	0.041	0.037 ± 0.029	0.313 ± 0.070	0.109	0.249 ± 0.100	0.961 ± 0.039	0.000	0.115 ± 0.089	0.671 ± 0.066	0.005

*K*^*trans*^ The volume transfer constant of contrast agent from a plasma space to an extravascular extracellular space (EES), *Ve* volume of extravascular extracellular space (EES) per unit volume of tissue

**Fig. 7:**
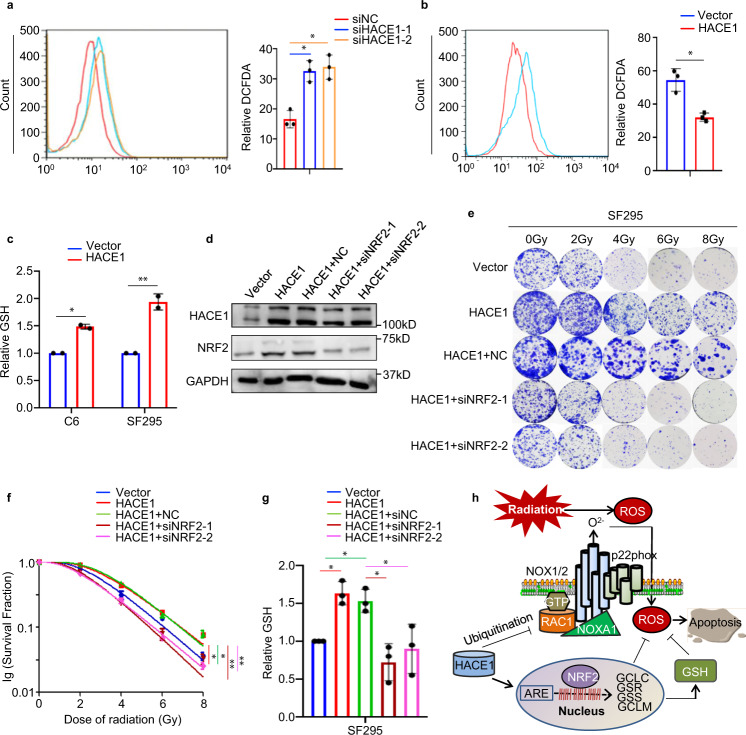
HACE1-mediated NRF2 activation reduces radiosensitivity of glioma cells by elevating cellular GSH content and reducing ROS content. **a** The effect of HACE1 knockdown in SF295 cells on cellular ROS levels was measured by CM-H2-DCFDA. **b** HACE1 overexpression-SF295 cells and control cells were treated with a 4 Gy X-ray. ROS production was then analyzed by DCF staining assay 24 h post-radiation. Left panel: the fluorescence intensity of ROS-positive cells. Right panel: the bar graph showing mean ± SD. **c** Measurement of cellular GSH content in HACE1 overexpression-C6 or SF295 cells and control cells. **d** Western blot analysis of HACE1, NRF2, and GAPDH (loading control) in SF295 cells with the indicated treatments. **e**, **f** The effect of NRF2 knockdown on the response of SF295 cells expressing HACE1 to radiation. **g** The effect of NRF2 knockdown in HACE1 overexpression-SF295 cells on cellular GSH levels. **h** A schematic model of HACE1 reducing radiosensitivity of glioma cells by regulating cellular ROS levels. The data were expressed as mean ± SD. *P < 0.05; **P < 0.01

It is the fact that NRF2 maintains cellular redox balance by mediating antioxidant response,^27^ and has been evidenced to cause radiation-resistance of cancer cells.^28,29^ We thus speculate that HACE1 decreases the radiosensitivity of glioma cells by activating NRF2. First, we measured cellular ROS content in SF295 cells by CM-H2-DCFDA, and demonstrated that HACE1 knockdown substantially increased cellular ROS levels compared with the control (Fig. 7a), while ectopic expression of HACE1 reduced cellular ROS levels (Fig. 7b).

Glutathione (GSH) usually inactivates endogenous ROS in mammalian cells, preventing ROS-related reactions triggered by external insults.^30^ Moreover, there are studies demonstrating that NRF2 disruption decreases GSH levels and increases ROS levels, thereby causing alveolar epithelial cell growth arrest.^31^ As supported, our results expectedly showed that HACE1 overexpression elevated GSH levels in C6 and SF295 cells (Fig. 7c). Meanwhile, our data showed that NRF2 knockdown could reverse HACE1-mediated decreased radiosensitivity of SF295 cells (Fig. 7d–f and Supplementary Table S3). On the contrary, when NRF2 was ectopically expressed in HACE1-deficient SF295 cells, the response of these cells to radiation was observably decreased (Supplementary Fig. S15 and Supplementary Table S4), strongly supporting the above conclusions. In addition, we found that NRF2 knockdown in SF295 cells effectively attenuated promoting effect of HACE1 on cellular GSH content (Fig. 7g).

Summarizing the above results, we have proposed a model to explicit the mechanism of HACE1 decreasing the radiosensitivity of glioma cells (Fig. 7h). Briefly, HACE1 targets complex-bound RAC1 and leads to RAC1 ubiquitination and degradation, thereby reducing cellular ROS levels.^12^ On the other hand, HACE1 increases GSH levels or upregulates NRF2 target genes by enhancing protein stability and IRES-mediated mRNA translation of NRF2, leading to decreased cellular ROS levels. These will ultimately cause decreased radiosensitivity of glioma cells.

## Discussion

HACE1 is often considered as a putative tumor suppressor involved in different types of cancer,^6^ while there is only one study revealing its pro-invasive role on melanoma cells.^14^ The present study provided convincing evidence showing its oncogenic role in glioma cells. We first observed elevated expression of HACE1 in gliomas compared with normal brain tissues, and a close correlation between high HACE1 expression and poor prognosis in patients with WHO grade III and IV. Second, by a series of functional experiments, we demonstrated that HACE1 exerted an oncogenic function in glioma cells through enhancing malignant behaviors of glioma cells in an E3 ligase-independent manner.

Considering that HACE1 is able to activate NRF2, a key oncoprotein in glioma, under oxidative stress,^17,32^ thus we determined the regulatory effect of HACE1 on NRF2 activity, attempting to illustrate its oncogenic role in glioma cells. Our data showed that HACE1 post-transcriptionally upregulated NRF2 expression via an E3 ligase-independent mechanism, and demonstrated that HACE1 promoted malignant behaviors of glioma cells through activating NRF2. As supported, a previous report indicated that HACE1 promoted NRF2 protein stabilization and synthesis under oxidative stress.^13^ However, the exact mechanism still remains a mystery. To address this, we first validated the effect of HACE1 on NRF2 protein stability. The results showed that HACE1 expectedly enhanced NRF2 protein stability. Further studies found that HACE1 interacted with and stabilized NRF2 by attenuating its ubiquitin degradation via competitively binding to NRF2 with KEAP1.

There is evidence showing that RNA binding protein La/SSB interacts with ribosome and enhances the recruitment of mRNAs containing a functional IRES element onto the ribosomes to drive de novo NRF2 protein translation.^23^ Thus, we hypothesize that HACE1 promotes IRES-mediated mRNA translation of NRF2 via La/SSB. As supported, our data showed that overexpression of HACE1 or HACE1_C876S_ enhanced *NRF2* 5′-UTR activity via an IRES element, and demonstrated that this effect was dependent on La/SSB. Notably, HACE1 not only increased La/SSB expression, but also impaired its nuclear/cytoplasm distribution, as supported by our data that HACE1 interacted and co-localized mostly with La/SSB in the extranuclear region of glioma cells.

We next expected to address the mechanism of HACE1 upregulating La/SSB. It is clear that La/SSB associates with nascent pri-miRNA transcripts in the nucleus and is required for the stability of some pri-miRNAs to process into pre-miRNAs. In addition, La/SSB can also protect pre-miRNAs from degradation by other nucleases.^33^ The present study demonstrated that some miRNAs such as miR-129-5p and miR-15b targeting La/SSB were downregulated when La/SSB was knocked down or HACE1/ HACE1_C876S_ was ectopically expressed in glioma cells. These results indicate that HACE1 increases La/SSB expression at post-transcriptional levels by reducing its nuclear localization and subsequently decreasing the expression of some miRNAs targeting La/SSB. Collectively, our data demonstrate that HACE1 enhances IRES-mediated mRNA translation of NRF2 through upregulating La/SSB at post-transcriptional levels, and this effect is similarly independent of its E3 ligase activity.

Through clinical data analysis, we speculate that HACE1 may affect the radiosensitivity of glioma patients. This was supported by our data that HACE1 overexpression decreased the sensitivity of glioma cells to radiotherapy compared to the control. HACE1 has been evidenced as a negative regulator of RAC1-dependent NADPH oxidase complexes, protecting cells from ROS-triggered DNA damage.^9^ The present study validated that HACE1 not only decreased cellular ROS levels, but also increased GSH levels, further supporting the above observations. In general, NRF2 is dysregulated in many tumors, thereby leading to the radioresistance during cancer therapy.^34^ Moreover, targeting NRF2 has been validated to be an exciting strategy for the radiosensitization by increasing ROS generation.^35,36^ Thus, we suppose that HACE1 decreases the radiosensitivity of glioma cells probably via NRF2 activation. Indeed, our data demonstrated that NRF2 knockdown effectively reversed HACE1-mediated decreased radiosensitivity of glioma cells and promoting effect of HACE1 on cellular GSH levels, strongly supporting the above hypothesis.

**Fig. 8:**
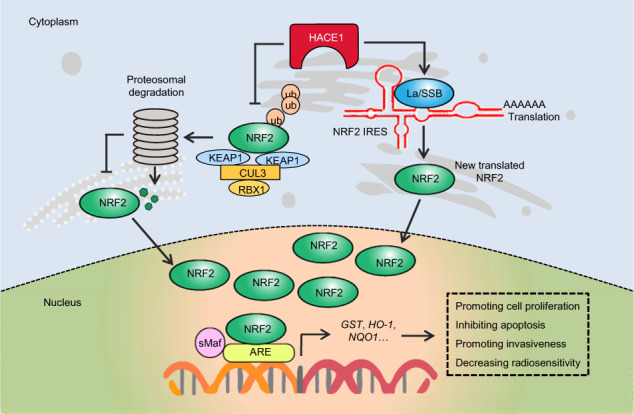
A schematic model for HACE1 promoting malignant phenotypes and reducing radiosensitivity of glioma cells. Briefly, increased expression of HACE1 enhances protein stability of NRF2 through competitively binding to NRF2 with another E3 ligase KEAP1. On the other hand, HACE1 promotes IRES-mediated mRNA translation of NRF2. HACE1 thus contributes to malignant behaviors and decreased radiosensitivity of glioma cells by activating NRF2

In summary, the present study demonstrates the oncogenic role of HACE1 in glioma cells, and determines that HACE1 causes enhanced malignant phenotypes and decreased radiosensitivity of glioma cells by enhancing protein stability and IRES-mediated mRNA translation of NRF2 (Fig. 8). Thus, our results support the notions that HACE1 is considered as a prognostic factor and potential therapeutic target in glioma, and targeting HACE1 may be therapeutically beneficial in glioma patients.

## Materials and methods

### Clinical samples

With the institutional review board approval and patient consent, we obtained 4 paraffin-embedded and 4 fresh-frozen normal brain tissues (from the patients with cerebral contusion and laceration) as well as 9 paraffin-embedded and 9 fresh-frozen gliomas (both portions are derived from different samples) from the First Affiliated Hospital of Xi’an Jiaotong University. All patients did not receive any therapies before surgery, and the tissues were histologically diagnosed by a senior pathologist at the Department of Pathology of the Hospital. Their clinicopathological data were summarized in Supplementary Table S5.

### Cell culture and drug treatments

Human glioma cell lines U87, SF295, and U251 were purchased from the American Type Culture Collection (ATCC) (Manassas, VA, USA) and the Cell Bank of Animal Laboratory Center of Zhongshan University (Guangzhou, China), respectively. The rat glioblastoma cell line C6 was purchased from Runying Biology Co., Ltd. U87, SF295 and U251 were routinely cultured at 37 °C in a DMEM medium with 10% fetal bovine serum (FBS), while C6 cells were cultured in RPMI 1640 medium with 10% FBS.

In some experiments, cells were treated with 10 μM CHX (MP Biomedicals, OH, USA) to block de novo protein synthesis. Moreover, cells were also treated with 25 μM proteasome inhibitor MG132 (Selleck Chemicals, TX, USA) to block ubiquitin-proteasomal degradation. The same volume of the vehicle was used as the control.

### RNA extraction and quantitative RT-PCR (qRT-PCR)

RNA isolation, reverse transcription, and qRT-PCR were performed according to a previous study.^37^ The primer sequences were shown in Supplementary Tables S6–8.

### Immunohistochemistry (IHC)

The expression of HACE1, NRF2, and Ki-67 in glioma tissues was evaluated by IHC using HACE1 antibody (Abcam), NRF2 antibody (Santa Cruz Biotechnology, Inc.), and Ki-67 antibody (BD Pharmingen). The detailed protocol was similarly described according to a previous study.^38^ Sections were then analyzed by a Tissue FAXS system (Tissuegnostics USA, Tarzana, CA, USA), and positive cells were directly counted using HistoQuest cytometry software according to the principle of flow cytometry.^39^

### siRNA, miRNAs, and expression plasmids

Target-specific siRNAs and control siRNA were purchased from Gene Pharma (Shanghai, China) and the sequences were shown in Supplementary Table S9. Cells were transfected with 50 nM siRNAs at ~70% confluence using Lipofectamine 2000 (Invitrogen, Grand Island, NY). One or two siRNAs with maximal knockdown efficiency were selected for the following experiments.

miRNA mimics and negative controls were purchased from RiboBio (Guangzhou, China) and the sequences were shown in Supplementary Table S10. In brief, cells were cultured in 6-well plates at ~60% confluence 24 h prior to transfection. miRNA mimics and negative controls at a final concentration of 30 nM were used for each transfection.

The plasmids expressing wild-type HACE1 and the enzymatically inactive HACE1 mutant HACE1_C876S_ were kindly provided by Prof. Ronggui Hu (Institute of Biochemistry and Cell Biology, Chinese Academy of Sciences). The primers used for plasmid construction were presented in Supplementary Table S11. The plasmid expressing NRF2 was purchased from Miao Ling Biotechnology Co., Ltd (Wuhan, China). Lentiviruses encoding HACE1/HACE1_C876S_ and control lentivirus were purchased from Hanbio Biotechnology Co., Ltd (Shanghai, China). Cell transfection was performed according to a previous study.^40^

### Western blot analysis

Western blot analysis was performed according to a previous protocol.^41^ The antibody information was shown in Supplementary Table S12.

### Preparation of nuclear and cytosolic fractions

Nuclear and cytosolic fractions were prepared by the Nuclear and Cytoplasmic Protein Extraction Kit (Beyotime, Beijing, China) according to the manufacturer’s instruction.

### In vitro functional studies

Cell proliferation was monitored by the iCELLigence real-time cell analysis system as described previously,^42^ and the data were expressed as normalized cell index (NCI) values. Besides, the protocols for cell viability, soft-agar colony formation, cell cycle distributions, cell apoptosis and migration/invasion assays were similarly described according to a previous study.^43^

### Co-immunoprecipitation (Co-IP) and immunofluorescence assays

The produces were similarly performed according to a previous study.^44^

### Dual-luciferase reporter assay

The protocol was described according to a previous study.^45^ The primer sequences used for the construction of dual-luciferase reporter plasmids were shown in Supplementary Table S13. The plasmid expressing mutant *La/SSB* 3′-UTR was synthesized by Tsingke Biotechnology Co. Ltd. (Beijing, China).

### In vivo tumorigenicity

The athymic nude mice (4- to 5-weeks-old) were obtained from SLAC Laboratory Animal Co., Ltd. (Shanghai, China), and randomly divided into two groups (*n* = 5/group). Next, we established a tumor xenograft model by subcutaneously injecting 8 × 10^6^ HACE1/HACE1_c876s_ overexpression-C6 or U87 cells or control cells into the right armpit region of nude mice. We then measured tumor size every 2 days using calipers from day 3 post-injection, and calculated tumor volumes according to the formula (length × width^2^ × 0.5). After 15–17 days, the mice were sacrificed, and tumors were isolated and weighted. A part of tumor tissues was then embedded in paraffin for subsequent IHC assays, and the remaining tumor tissues were used for western blot analysis. All animal experiments were approved by the Animal Ethics Committee of Xi’an Jiaotong University.

### X-ray radiation treatment

Glioma cells were treated with 0, 2, 4, 6, 8 Gy of X-ray by a linear accelerator (Clinac 2100EX, Varian Medical Systems). Colony formation assay was performed according to a previous protocol.^46^

### MRI protocol and data post-processing

Male Sprague-Dawley rats (*n* = 6; 240–270 g) were anesthetized, given analgesic, and inoculated with 1 × 10^5^ C6 cells via stereotaxic injection. All rats were similarly evaluated by conventional and Dynamic Contrast-Enhanced MRI (DCE-MRI) before and after radiation as described previously.^47,48^ With each tumor, 10–15 regions of interest (ROIs) were manually placed on TP and PT area. Next, we calculated permeability parameters (such as *K*^trans^ and Ve), assessed their diagnostic efficiency, transferred all DCE-MRI data to the post-processing workstation, and analyzed related data by a commercial software tool (OK; GE Healthcare). The above procedures were approved by Xi’an Jiaotong University’s Institutional Animal Care and Use Committee.

### Measurement of cellular ROS

Cells were incubated with 5 mM 2′7′-dichlorodihydrofluorescein diacetate (DCFDA) (Invitrogen, CA, USA) at 37 °C for 20 min, washed twice in PBS, and trypsinized. The fluorescence was then measured by flow cytometry.

### Redox-state analysis

GSH levels were measured by a GSH and GSSG Assay Kit (Beyotime, Beijing, China) according to a previous study.^49^

### Statistical analysis

SPSS statistical package (16.0, Chicago, IL) was used to compare gene expression difference between tumor tissues and control subjects by two-tailed *t*-test and Mann–Whitney *U* test. The data are expressed as mean ± standard deviation (SD). *P* < 0.05 was considered statistically significant.

## Supplementary information

Supplementary Materials

## Data Availability

The authors confirm that the data supporting the findings of this study are available within the article and its Supplementary Materials.

## References

[1] Louis DN (2016). The 2016 World Health Organization Classification of tumors of the central nervous system: a summary. Acta Neuropathol..

[2] Stewart LA (2002). Chemotherapy in adult high-grade glioma: a systematic review and meta-analysis of individual patient data from 12 randomised trials. Lancet.

[3] Yan Y (2019). Novel function of lncRNA ADAMTS9-AS2 in promoting temozolomide resistance in glioblastoma via upregulating the FUS/MDM2 ubiquitination axis. Front. Cell Dev. Biol..

[4] Yan Y (2019). FGFR2-mediated Phosphorylation of PTEN at Tyrosine 240 Contributes to the Radioresistance of Glioma. J. Cell Commun. Signal..

[5] Slade I (2010). Constitutional translocation breakpoint mapping by genome-wide paired-end sequencing identifies HACE1 as a putative Wilms tumour susceptibility gene. J. Med. Genet..

[6] Zhang L (2007). The E3 ligase HACE1 is a critical chromosome 6q21 tumor suppressor involved in multiple cancers. Nat. Med..

[7] Torrino S (2011). The E3 ubiquitin-ligase HACE1 catalyzes the ubiquitylation of active Rac1. Dev. Cell..

[8] Goka ET, Lippman ME (2015). Loss of the E3 ubiquitin ligase HACE1 results in enhanced Rac1 signaling contributing to breast cancer progression. Oncogene.

[9] Daugaard M (2013). Hace1 controls ROS generation of vertebrate Rac1-dependent NADPH oxidase complexes. Nat. Commun..

[10] Castillo-Lluva S (2013). The tumour suppressor HACE1 controls cell migration by regulating Rac1 degradation. Oncogene.

[11] Cheng G, Diebold BA, Hughes Y, Lambeth JD (2006). Nox1-dependent reactive oxygen generation is regulated by Rac1. J. Biol. Chem..

[12] Ueyama T, Geiszt M, Leto TL (2006). Involvement of Rac1 in activation of multicomponent Nox1- and Nox3-based NADPH oxidases. Mol. Cell Biol..

[13] Liu Z (2014). Ubiquitylation of autophagy receptor Optineurin by HACE1 activates selective autophagy for tumor suppression. Cancer Cell..

[14] El-Hachem N (2018). Uncovering and deciphering the pro-invasive role of HACE1 in melanoma cells. Cell Death Differ..

[15] Giese A, Bjerkvig R, Berens ME, Westphal M (2003). Cost of of migration: invasion of malignant gliomas and implications for treatment. J. Clin. Oncol..

[16] Nagendra K (2016). Low doses of PEG-coated gold nanoparticles sensitize solid tumors to cold plasma by blocking the PI3K/AKT-driven signaling axis to suppress cellular transformation by inhibiting growth and EMT. Biomaterials.

[17] Rotblat B (2014). HACE1 reduces oxidative stress and mutant Huntingtin toxicity by promoting the NRF2 response. Proc. Natl Acad. Sci. USA.

[18] Sukumari-Ramesh S, Prasad N, Alleyne CH, Vender JR, Dhandapani KM (2015). Overexpression of Nrf2 attenuates Carmustine-induced cytotoxicity in U87MG human glioma cells. BMC Cancer.

[19] Lu DY (2012). Osteopontin increases heme oxygenase-1 expression and subsequently induces cell migration and invasion in glioma cells. Neuro-Oncol..

[20] Ohta T (2008). Loss of Keap1 function activates Nrf2 and provides advantages for lung cancer cell growth. Cancer Res..

[21] Li W (2010). An internal ribosomal entry site mediates redox-sensitive translation of Nrf2. Nucleic Acids Res..

[22] Hellen CU, Sarnow P (2001). Internal ribosome entry sites in eukaryotic mRNA molecules. Genes Dev..

[23] Zhang J, Dinh TN, Kappeler K, Tsaprailis G, Chen QM (2012). La autoantigen mediates oxidant induced de novo Nrf2 protein translation. Mol. Cell Proteom..

[24] Kapsogeorgou EK, Gourzi VC, Manoussakis MN (2011). Cellular microRNAs (miRNAs) and Sjogren’s syndrome: candidate regulators of autoimmune response and autoantigen expression. J. Autoimmun..

[25] Knitter JR (2018). Interval change in diffusion and perfusion MRI parameters for the assessment of pseudoprogression in cerebral metastases treated with stereotactic radiation. AJR.

[26] Yun TJ (2015). Glioblastoma treated with concurrent radiation therapy and temozolomide chemotherapy differentiation of true progression from pseudoprogression with quantitative dynamic contrast-enhanced MR imaging. Radiology.

[27] Nguyen T, Nioi P, Pickett CB (2009). The Nrf2-antioxidant response element signaling pathway and its activation by oxidative stress. J. Biol. Chem..

[28] McDonald JT (2010). Ionizing radiation activates the Nrf2 antioxidant response. Cancer Res..

[29] Wang T (2017). Role of Nrf2 signaling pathway in the radiation tolerance of patients with head and neck squamous cell carcinoma: an in vivo and in vitro study. Onco. Targets Ther..

[30] Thangamani S (2017). Ebselen exerts antifungal activity by regulating glutathione (GSH) and reactive oxygen species (ROS) production in fungal cells. Biochim. Biophys. Acta Gen. Subj..

[31] Roh JL, Kim EH, Jang H, Shin D (2017). Nrf2 inhibition reverses the resistance of cisplatin-resistant head and neck cancer cells to artesunate-induced ferroptosis. Redox Biol..

[32] Zhu J (2013). Nrf2 is required to maintain the self-renewal of glioma stem cells. BMC Cancer.

[33] Liang C (2013). Sjogren syndrome antigen B (SSB)/La promotes global microRNA expression by binding microRNA precursors through stem-loop recognition. J. Biol. Chem..

[34] Jayakumar S, Kunwar A, Sandur SK, Pandey BN, Chaubey RC (2014). Differential response of DU145 and PC3 prostate cancer cells to ionizing radiation: role of reactive oxygen species, GSH and Nrf2 in radiosensitivity. Biochim. Biophys. Acta.

[35] Zhou S, Ye W, Shao Q, Zhang M, Liang J (2013). Nrf2 is a potential therapeutic target in radioresistance in human cancer. Crit. Rev. Oncol. Hematol..

[36] Sekhar KR, Freeman ML (2015). Nrf2 promotes survival following exposure to ionizing radiation. Free Radic. Biol. Med..

[37] Ma X, Wang M, Yin T, Zhao Y, Wei X (2019). Myeloid-derived suppressor cells promote metastasis in breast cancer after the stress of operative removal of the primary cancer. Front. Oncol..

[38] Shi J (2016). Increased expression of EHF via gene amplification contributes to the activation of HER family signaling and associates with poor survival in gastric cancer. Cell Death Dis..

[39] Jason KS (2020). Transcriptional regulatory networks of tumor-associated macrophages that drive malignancy in mesenchymal glioblastoma. Genome Biol..

[40] Li Y (2018). ZNF677 suppresses Akt phosphorylation and tumorigenesis in thyroid cancer. Cancer Res..

[41] Li Y (2018). c-Myc is a major determinant for antitumor activity of aurora A kinase inhibitor MLN8237 in thyroid cancer. Thyroid.

[42] Prudnikova TY, Villamar-Cruz O, Rawat SJ, Cai KQ, Chernoff J (2016). Effects of p21-activated kinase 1 inhibition on 11q13-amplified ovarian cancer cells. Oncogene.

[43] Wei J (2019). Increased expression of NAF1 contributes to malignant phenotypes of glioma cells through promoting protein synthesis and associates with poor patient survival. Oncogenesis.

[44] Zhang Z (2019). Repurposing brigatinib for the treatment of colorectal cancer based on inhibition of ER-phagy. Theranostics.

[45] Dang S (2019). Dynamic expression of ZNF382 and its tumor-suppressor role in hepatitis B virus-related hepatocellular carcinogenesis. Oncogene.

[46] Xi R (2016). HPV16 E6-E7 induces cancer stem-like cells phenotypes in esophageal squamous cell carcinoma through the activation of PI3K/Akt signaling pathway in vitro and in vivo. Oncotarget.

[47] Hormuth DA, Skinner JT, Does MD, Yankeelov TE (2014). A comparison of individual and population-derived vascular input functions for quantitative DCE-MRI in rats. Magn. Reson. Imaging.

[48] Zhao J, Yang ZY, Luo BN, Yang JY, Chu JP (2015). Quantitative evaluation of diffusion and dynamic contrast-enhanced MR in tumor parenchyma and peritumoral area for distinction of brain tumors. PLoS ONE.

[49] Xie JM (2014). TIGAR has a dual role in cancer cell survival through regulating apoptosis and autophagy. Cancer Res..

