# Supplementary material for: HACE1-mediated NRF2 activation causes enhanced malignant phenotypes and decreased radiosensitivity of glioma cells
Source: Signal Transduct Target Ther. 2021 Nov 24;6:399. doi: 10.1038/s41392-021-00793-z (PMC8611003; doi:10.1038/s41392-021-00793-z)
Supplement: Supplementary file 1 — Supplementary Materials [file 41392_2021_793_MOESM1_ESM.docx]

Supplementary Materials for

**HACE1-mediated NRF2 activation causes enhanced malignant phenotypes and decreased radiosensitivity of glioma cells**

Chenxing Da^1,5^, Jun Pu^1^, Zhe Liu^2^, Jing Wei^1^, Yiping Qu^1^, Yongxing Wu^3^, Bingyin Shi^1^, Jian Yang^2,*^, Nongyue He^4,*^, and Peng Hou^1,*^

^1^Key Laboratory for Tumor Precision Medicine of Shaanxi Province and Department of Endocrinology, The First Affiliated Hospital of Xi’an Jiaotong University, Xi’an 710061, P.R. China

2Department of Diagnostic Radiology, The First Affiliated Hospital of Xi’an Jiaotong University, Xi’an 710061, P.R. China

3Department of Neurosurgery, The First Affiliated Hospital of Xi’an Jiaotong University, Xi’an 710061, P.R. China

4State Key Laboratory of Bioelectronics, Southeast University, Nanjing 210096, P.R. China

5Shanxi Provincial Crops Hospital of Chinese People’s Armed Police Force, Xi’an 710054, P.R. China

***Correspondence to:**

E-mail (Peng Hou): [phou@xjtu.edu.cn](mailto:phou@xjtu.edu.cn)

E-mail (Nongyue He): nyhe1958@163.com

E-mail (Jian Yang): [yj1118@mail.xjtu.edu.cn](mailto:yj1118@mail.xjtu.edu.cn)

**Supplementary Materials for this manuscript include the following:**

Fig. S1. HACE1 promotes the viability and colony formation of SF295 and U87 cells.

Fig. S2. HACE1 promotes the growth of C6 cells in vitro and in vivo.

Fig. S3. Ki-67 staining of xenograft tumors.

Fig. S4. HACE1 promotes glioma cancer cell invasiveness.

Fig. S5. High NRF2 expression is associated with poor survival in glioma patients.

Fig. S6. The effects of ectopic expression of HACE1 or HACE1C876S in SF295 cells on mRNA expression of NRF2 target genes HMOX1 and NQO1 were determined by qRT-PCR.

Fig. S7. The expression of HACE1 and NRF2 proteins in the representative xenograft tumors was analyzed by western blot analysis and measured using densitometry.

Fig. S8. NRF2 knockdown attenuates promoting effect of HACE1 on glioma cell proliferation and invasiveness.

Fig. S9. Ectopic expression of NRF2 reverses inhibitory effect of HACE1 knockdown on glioma cell proliferation and invasiveness.

Fig. S11. La/SSB was ectopically expressed in U87 and SF295 cells knocking down HACE1.

Fig. S12. HACE1 enhances the activity of La/SSB 3’-UTR.

Fig. S13. HACE1 enhances the growth of C6 cells in an orthotopic rat glioma model.

Fig. S14. Change ratio of tumor volume in the indicated rats before and after radiation.

Fig. S15. HACE1-mediated NRF2 activation promotes radioresistance of glioma cells by increasing cellular GSH levels.

Table S1. Constant values of radiation biology in SF295 cells.

Table S2. Constant values of radiation biology in C6 cells

Table S3. Constant values of radiation biology in SF295 cells

Table S4. Constant values of radiation biology in SF295 cells

Table S5. Clinicopathological data in glioma patients.

Table S6. qRT-PCR primer sequences used in this study for the indicated genes

Table S7. miRNA-specific RT primer sequences

Table S8. qRT-PCR primer sequences used in this study for the indicated miRNAs

Table S9. The sequences of siRNAs used in this study

Table S10. miRNA mimics and negative controls used in this study

Table S11. The primers used in this study for plasmid construction

Table S12. The antibodies used in this study

Table S13. The primers used in this study for luciferase reporter plasmid construction

**Fig. S1. HACE1 promotes the viability and colony formation of SF295 and U87 cells.** **a** The MTT assays were performed to monitor the effects of HACE1 knockdown on cell viability in SF295 and U87 cells. **b** HACE1 was knocked down in the indicated cells, and colony size and number were quantified by ImageJ analysis software. **c** The MTT assays were performed to monitor the effects of ectopic expression of HACE1 or HACE1_C876S_ on cell viability in SF295 and U87 cells. **d** HACE1 or HACE1_C876S_ was ectopically expressed in the indicated cells, and colony size and number were quantified by ImageJ analysis software. The data were shown as mean ± SD. *, *P* < 0.05. **, *P* < 0.01.

**Fig. S2. HACE1 promotes the growth of C6 cells *in vitro* and *in vivo*.** **a** The MTT assay and iCELLigence system were used to monitor the effect of ectopic expression of HACE1 and HACE1_C876S_ on the proliferation of C6 cells. **b** Tumor growth curves in nude mice were compared between C6 cells stably expressing HACE1 or HACE1_C876S_ and control cells. Data were presented as mean ± SD (n = 4/group). **c** Representative pictures of xenograft tumors (left panel) and statistical results of tumor weight (right panel) from HACE1-overexpression and control groups. The data were shown as mean ± SD. *, *P* < 0.05; **, *P* < 0.01**.**

**Fig. S3. Ki-67 staining of xenograft tumors.** **a, b** Shown is representative HACE1 and Ki-67 staining in U87/C6 cell-derived xenograft tumors from HACE1/HACE1C876S -overexpression and control mice (left panels). Histogram represents mean ± SD of the percentage of HACE1/Ki-67 positive cells from five microscopic fields in each group (right panels). Scale bar, 200 μm. *, *P* < 0.05; **, *P* < 0.01.

**
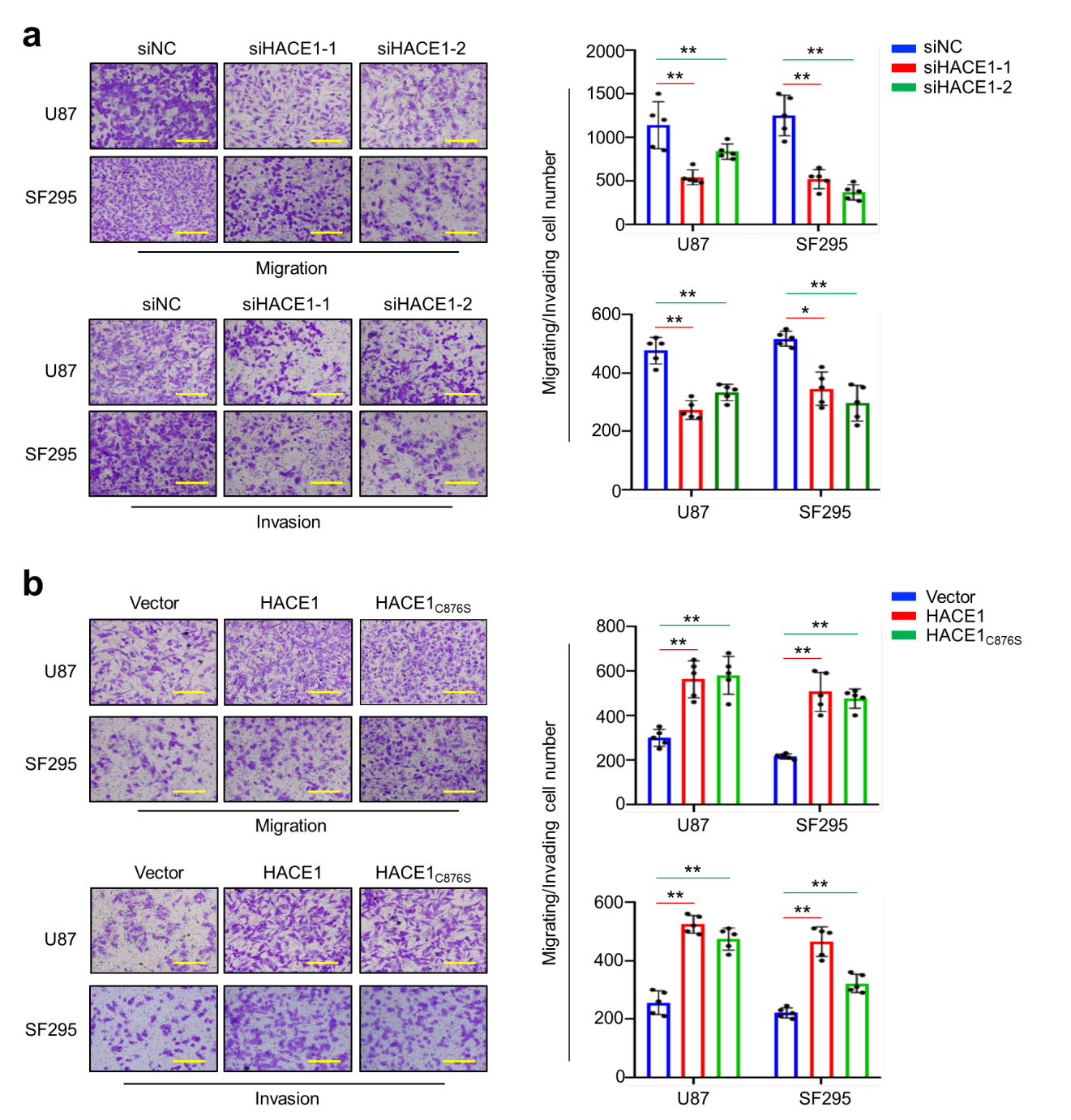
**

**Fig. S4. HACE1 promotes glioma cancer cell invasiveness.** **a** The effect of HACE1 knockdown on migration and invasion potential of U87 and SF295 cells. **b** The effect of ectopic expression of HACE1 and HACE1_C876S_ on migration and invasion potential of U87 and SF295 cells. The representative pictures of migrated/invaded cells were shown in the left panels, and statistical data of cell numbers from three different experiments were shown in the right panels. The data were shown as mean ± SD. *, *P* < 0.05; **, *P* < 0.01.

**Fig. S5. High NRF2 expression is associated with poor survival in glioma patients.** **a** Immunohistochemistry (IHC) was performed to assess NRF2 expression in gliomas (n =9) and normal brain tissues (n =4). Shown in left panels is representative NRF2 staining. Scale bar, 200 μm. Sections were analyzed by a Tissue FAXS system (Tissuegnostics USA, Tarzana, CA, USA), and positive cells were counted using HistoQuest cytometry software (left panel). **b** NRF2 expression was determined in gliomas (n =9) and normal brain tissues (n =4) by western blot analysis. GAPDH was used as a loading control. The densitometry analysis was performed to measure the levels of NRF2 proteins on the western blot (left panel), and statistical results were presented in the right panels. **c** The association of *NRF2* expression with poor survival in glioma patients (data from TCGA dataset). The data were shown as mean ± SD. **, *P* < 0.01.

**Fig. S6.** The effects of ectopic expression of HACE1 or HACE1_C876S_ in SF295 cells on mRNA expression of NRF2 target genes *HMOX1* and *NQO1* were determined by qRT-PCR. *18S* rRNA were used as a normalized control. The data were shown as mean ± SD. *, *P* < 0.05, **, *P* < 0.01.

**Fig. S7.** The expression of HACE1 and NRF2 proteins in the representative xenograft tumors was analyzed by western blot analysis and measured using densitometry. GAPDH was used as a loading control. The data were shown as mean ± SD. *, *P* < 0.05, **, *P* < 0.01.

**
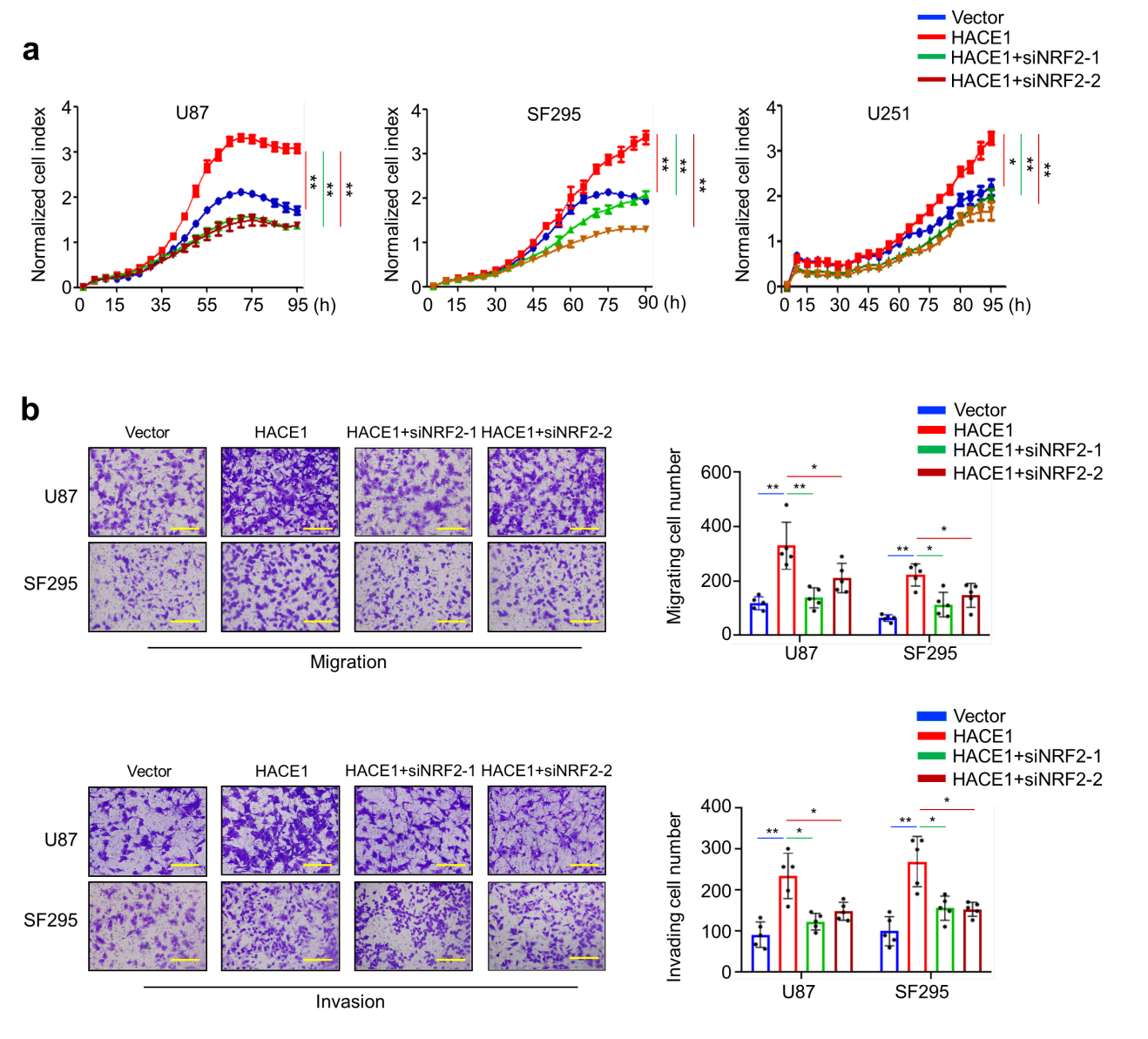
**

**Fig. S8. NRF2 knockdown attenuates promoting effect of HACE1 on glioma cell proliferation and invasiveness. a** NRF2 was knocked down in U87, SF295 and U251 cells stably expressing HACE1, and iCELLigence system was then used to monitor the proliferation of the indicated cells. **b** NRF2 was similarly knocked down in U87 and SF295 cells stably expressing HACE1, and transwell assays were then performed to evaluate migration or invasion potential of the indicated cells. The representative pictures of migrated/invaded cells were shown in the left panels, and statistical data of cell numbers from three different experiments were shown in the right panels. The data were shown as mean ± SD. *, *P* < 0.05; **, *P* < 0.01.

**
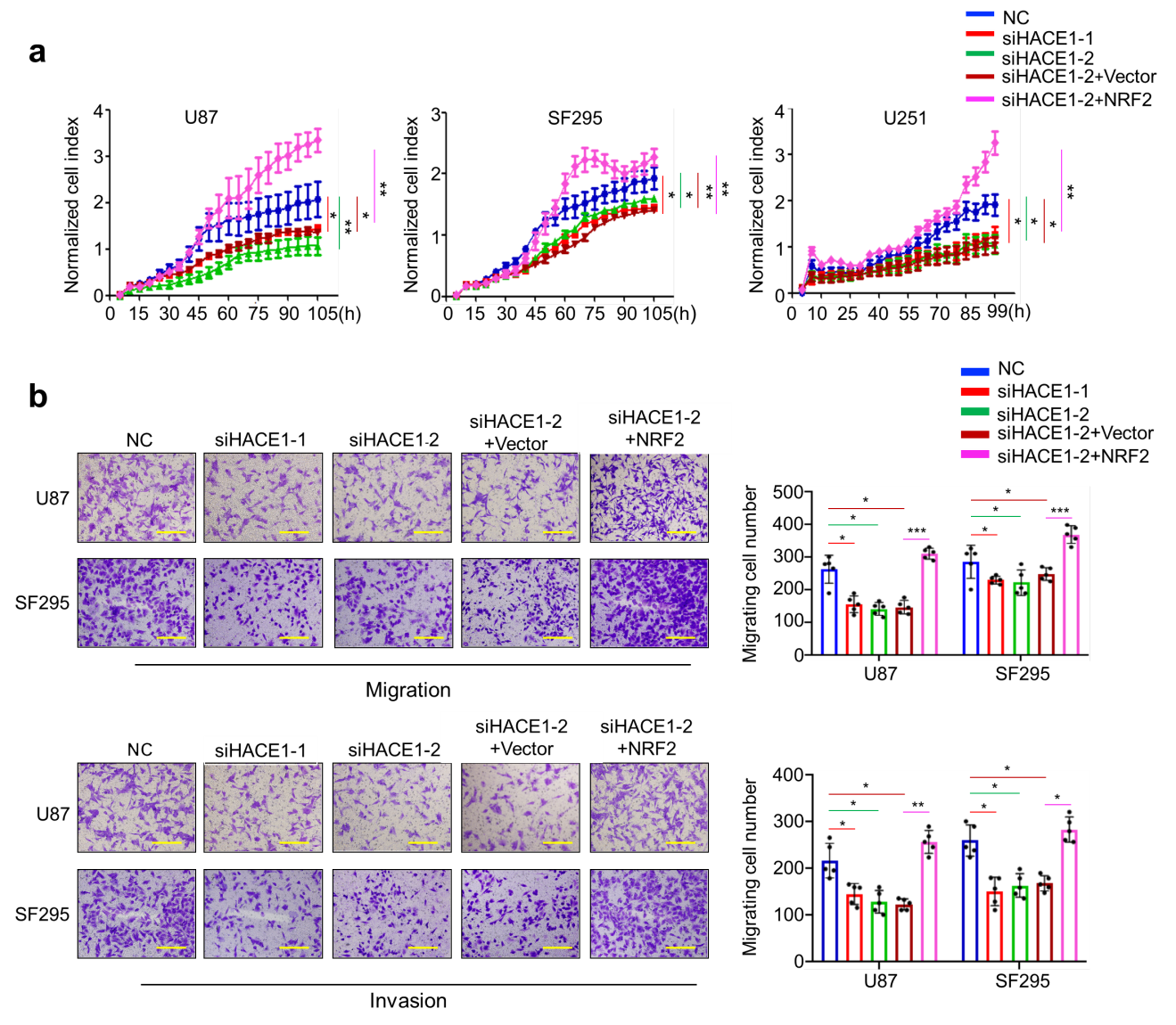
**

**Fig. S9. Ectopic expression of NRF2 reverses inhibitory effect of HACE1 knockdown on glioma cell proliferation and invasiveness. a** NRF2 was ectopically expressed in U87, SF295 and U251 cells knocking down HACE1, and iCELLigence system was then used to monitor the proliferation of the indicated cells. **b** NRF2 was similarly re-expressed in U87 and SF295 cells knocking down HACE1, and transwell assays were then performed to evaluate migration or invasion potential of the indicated cells. The representative pictures of migrated/invaded cells were shown in the left panels, and statistical data of cell numbers from three different experiments were shown in the right panels. The data were shown as mean ± SD. *, *P* < 0.05; **, *P* < 0.01; ***, *P* < 0.001.


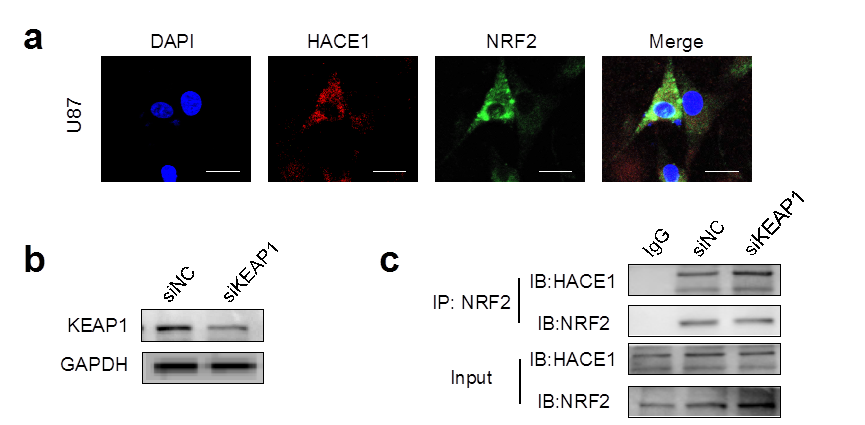


**Fig. S10.** **a** Immunofluorescence was performed to determine the co-localization of HACE1 and NRF2 in extranuclear region of SF295 and U87 cells. Blue color represents DAPI staining for nuclei; Red color represents HACE1; Green color represents NRF2. Scale bars, 20 μm. **b** Western blot was performed to validate KEAP1 knockdown in SF295 cells. GAPDH was used as a loading control. **c** Co-IP assay was performed to assess the effect of KEAP1 knockdown on HACE1-NRF2 interaction using the indicated antibodies.

**Fig. S11.** La/SSB was ectopically expressed in U87 and SF295 cells knocking down HACE1. The dual-luciferase reporter system was then performed to analyze the IRES activity of NRF2 in the indicated cells. The data were shown as mean ± SD. *, *P* < 0.05.

**Fig. S12. HACE1 enhances the activity of *La/SSB* 3^’-^UTR. a, b** HACE1 was knocked down or ectopically expressed in SF295 cells. mRNA expression of *La/SSB* was then measured by qRT-PCR assay. *18S* rRNA was used as a normalized control. **c** The effect of ectopic expression of HACE1 or HACE1_C876S_ on *La/SSB* 3^’-^UTR activity in SF295 cells was evaluated by the dual-luciferase reporter system. The ratio of the Luc/Renilla activity was evaluated in three independent assays. The data were presented as mean ± SD. *, *P* < 0.05; **, *P* < 0.01; ***, *P* < 0.001.

**Fig. S13.** **HACE1 enhances the growth of C6 cells in an orthotopic rat glioma model. a** Eighteen days after implantation of C6 cells, brains tissue of rats were removed by left ventricular perfusion, and coronal incision was then performed after fixation (left panels). H&E staining of a centrally located tissue slice is shown in right panels. Arrow represents tumor site. Scale bar, 200 μm. **b** Shown is representative HACE1, NRF2 and Ki-67 staining of xenograft tumors from HACE1-overexpression and control groups (left panels). Histogram represents mean ± SD of the percentage of positive cells from five microscopic fields in each group (right panel). Scale bar, 200 μm. The data were presented as mean ± SD. *, *P* < 0.05; **, *P* < 0.01.

**Fig. S14. Change ratio of tumor volume in the indicated rats before and after radiation.** Tumor volumes of two groups before and after radiation were measured by ImageJ [software](http://www.baidu.com/link?url=B4IdLEBRXfs39gX60tTqrH7mtNeGCWra9-bWoPPY7znm-307hvTK0idhbaE-JMsgLEQOFIzfQbaZHZxf5kx3QVY25Iz-CGl_v6NWnOLErrC). Data were shown as mean ± SD. **, *P* < 0.01.

**Fig. S15. HACE1-mediated NRF2 activation promotes radioresistance of glioma cells by increasing cellular GSH levels.** **a** NRF2 was ectopically expressed in SF295 cells knocking down HACE1. Western blot analysis was performed to analyze the expression of HACE1 and NRF2 in cells with the indicated treatments. GAPDH was used as a loading control. **b**, **c** The effect of ectopic expression of NRF2 on the response of SF295 cells knocking down HACE1 to radiation. **d** The effect of ectopic expression of NRF2 in SF295 cells knocking down HACE1 on cellular GSH levels. The data were presented as mean ± SD. *, *P* < 0.05; **, *P* < 0.01.

**Table S1**. Constant values of radiation biology in SF295 cells

|  | **D0** | ***P*** | **Dq** | | ***P*** | **k** | | ***P*** | **N** | ***P*** | **SF2(%)** | ***P*** | **SER(D0)** | **SER(Dq)** |
| --- | --- | --- | --- | --- | --- | --- | --- | --- | --- | --- | --- | --- | --- | --- |
| Vector | 4.442±0.512 |  | | 3.581±0.034 |  | 0.231±0.026 | |  | 2.302±0.196 |  | 84.2±1.195 |  |  |  |
| HACE1 | 7.268±0.690 | 0.030 | | 5.574±0.561 | 0.041 | 0.207±0.052 | 0.703 | | 3.090±0.541 | 0.243 | 92.29±2.797 | 0.056 | 0.575±0.029 | 0.566±0.011 |

Constant values of radiation biology were calculated based on the data of colony formation assay. D0 (D0 = 1/k) is the mean lethal dose which cells were hitted and causing death. Dq is the quasi-threshold dose which represents the shoulder of the dose-survival curve. The higher value of D0 and Dq represent the more radioresistance of cells. N is extrapolation number of the dose-survival curve which positive related with the repair ability of cells. SF2 is the survival fraction of cells after expose to 2 Gy ionizing radiation and represents the radioresistance of cells. SER reflects the difference and change of cell radiosensitivity. SER represents the radiosensitiveness of cells. All the data above are represented as mean ± S.D. of three independent experiment.

**Table S2**. Constant values of radiation biology in C6 cells

|  | **D0** | ***P*** | **Dq** | | ***P*** | **k** | | ***P*** | **N** | ***P*** | **SF2(%)** | ***P*** | **SER(D0)** | **SER(Dq)** |
| --- | --- | --- | --- | --- | --- | --- | --- | --- | --- | --- | --- | --- | --- | --- |
| Vector | 4.144±0.664 |  | | 3.858±0.117 |  | 0.253±0.039 | |  | 2.761±0.461 |  | 81.4±6.836 |  |  |  |
| HACE1 | 7.126±0.793 | 0.044 | | 6.812±0.118 | 0.000 | 0.171±0.031 | 0.177 | | 2.711±0.323 | 0.932 | 92.7±0.311 | 0.174 | 0.842±0.012 | 0.609±0.002 |

Constant values of radiation biology were calculated based on the data of colony formation assay. D0 (D0 = 1/k) is the mean lethal dose which cells were hitted and causing death. Dq is the quasi-threshold dose which represents the shoulder of the dose-survival curve. The higher value of D0 and Dq represent the more radioresistance of cells. N is extrapolation number of the dose-survival curve which positive related with the repair ability of cells. SF2 is the survival fraction of cells after expose to 2 Gy ionizing radiation and represents the radioresistance of cells. SER reflects the difference and change of cell radiosensitivity. SER represents the radiosensitiveness of cells. All the data above are represented as mean ± S.D. of three independent experiment.

**Table S3**. Constant values of radiation biology in SF295 cells

|  | **D0** | ***P*** | **Dq** | | ***P*** | **k** | | ***P*** | **N** | ***P*** | **SF2(%)** | ***P*** | **SER(D0)** | **SER(Dq)** |
| --- | --- | --- | --- | --- | --- | --- | --- | --- | --- | --- | --- | --- | --- | --- |
| Vector | 1.601±0.163 |  | | 2.489±0.115 |  | 0.629±0.063 | |  | 4.888±1.096 |  | 81.16±4.406 |  |  |  |
| HACE1 | 1.735±0.133 | 0.016 | | 3.012±0.089 | 0.001 | 0.579±0.044 | 0.046 | | 5.791±1.055 | 0.000 | 93.26±2.003 | 0.070 | 0.921±0.022 | 0.826±0.014 |
| HACE1+NC | 1.728±0.090 | 0.122 | | 3.200±0.067 | 0.001 | 0.579±0.029 | 0.157 | | 7.189±1.135 | 0.000 | 94.00±4.726 | 0.007 | 0.925±0.052 | 0.773±0.020 |
| HACE1+siNRF2-1 | 1.470±0.062 | 0.008 | | 1.938±0.068 | 0.000 | 0.618±0.049 | 0.370 | | 3.776±0.511 | 0.011 | 74.11±4.600 | 0.002 | 1.112±0.067 | 1.654±0.067 |
| HACE1+siNRF2-2 | 1.579±0.058 | 0.168 | | 1.784±0.104 | 0.002 | 0.633±0.023 | 0.207 | | 3.110±0.347 | 0.012 | 70.33±7.810 | 0.006 | 1.036±0.071 | 1.807±0.154 |

Constant values of radiation biology were calculated based on the data of colony formation assay. D0 (D0 = 1/k) is the mean lethal dose which cells were hitted and causing death. Dq is the quasi-threshold dose which represents the shoulder of the dose-survival curve. The higher value of D0 and Dq represent the more radioresistance of cells. N is extrapolation number of the dose-survival curve which positive related with the repair ability of cells. SF2 is the survival fraction of cells after expose to 2 Gy ionizing radiation and represents the radioresistance of cells. SER reflects the difference and change of cell radiosensitivity. SER represents the radiosensitiveness of cells. All the data above are represented as mean ± S.D. of three independent experiment.

**Table S4**. Constant values of radiation biology in SF295 cells

|  | **D0** | ***P*** | **Dq** | | ***P*** | **k** | | ***P*** | **N** | ***P*** | **SF2(%)** | ***P*** | **SER(D0)** | **SER(Dq)** |
| --- | --- | --- | --- | --- | --- | --- | --- | --- | --- | --- | --- | --- | --- | --- |
| NC | 1.582±0.060 |  | | 2.150±0.187 |  | 0.632±0.023 | |  | 3.898±0.338 |  | 78.67±1.528 |  |  |  |
| siHACE1-1 | 1.541±0.078 | 0.393 | | 1.489±0.136 | 0.002 | 0.649±0.033 | 0.046 | | 2.641±0.264 | 0.001 | 62.67±3.786 | 0.016 | 1.028±0.044 | 1.444±0.020 |
| siHACE1-2 | 1.568±0.037 | 0.709 | | 1.426±0.115 | 0.024 | 0.638±0.015 | 0.729 | | 2.485±0.140 | 0.022 | 62.00±3.00 | 0.002 | 1.009±0.037 | 1.047±0.107 |
| siHACE1-2+Vector | 1.533±0.0729 | 0.536 | | 1.600±0.063 | 0.017 | 0.653±0.031 | 0.530 | | 2.856±0.258 | 0.002 | 61.87±4.61 | 0.041 | 1.006±0.0514 | 0.892±0.083 |
| siHACE1-2+NRF2 | 1.746±0.045 | 0.005 | | 2.375±0.034 | 0.000 | 0.573±0.015 | 0.013 | | 3.907±0.215 | 0.000 | 77.27±1.650 | 0.032 | 0.898±0.018 | 0.673±0.017 |

Constant values of radiation biology were calculated based on the data of colony formation assay. D0 (D0 = 1/k) is the mean lethal dose which cells were hitted and causing death. Dq is the quasi-threshold dose which represents the shoulder of the dose-survival curve. The higher value of D0 and Dq represent the more radioresistance of cells. N is extrapolation number of the dose-survival curve which positive related with the repair ability of cells. SF2 is the survival fraction of cells after expose to 2 Gy ionizing radiation and represents the radioresistance of cells. SER reflects the difference and change of cell radiosensitivity. SER represents the radiosensitiveness of cells. All the data above are represented as mean ± S.D. of three independent experiment.

**Table S5.** Clinicopathological data in glioma patients

| **NO. of**  **patients** | **Gender** | **Age, years** | **Tumor localization** | **Histological types** | **WHO grade** |
| --- | --- | --- | --- | --- | --- |
| Paraffin-embedded tissues | | | | | |
| G3 | Male | 24 | Right frontotemporal | Astrocytoma | II |
| G4 | Female | 57 | Right frontal lobe | Astrocytoma | II |
| G5 | Male | 40 | Right parietal lobe | Astrocytoma oligodendrocyte | II |
| G6 | Male | 61 | Right frontal lobe | Astrocytoma | II |
| G9 | Female | 49 | Left cerebellum | Anaplastic astrocytoma | III |
| G12 | Male | 73 | Right parietal lobe | Anaplastic astrocytoma | III |
| G16 | Female | 30 | Right frontal lobe | Astrocytoma | II |
| G18 | Male | 68 | Vermis cerebelli | Astrocytoma | II |
| G17 | Female | 54 | Right frontotemporal | Astrocytoma | II |
| Fresh-frozen gliomas | |  |  |  |  |
| T1 | Female | 27 | Left parietal lobe | Astrocytoma | II |
| T4 | Female | 22 | cerebellum | Astrocytoma | II |
| T10 | Male | 61 | Right temporal lobe | Oligocytic astrocytoma | II |
| T11 | Female | 29 | Left thalamus | Astrocytoma | II |
| T14 | Male | 39 | [Right frontal lobe](https://dict.cn/Right%20frontal%20lobe) | [Anaplastic astrocytoma](https://dict.cn/Anaplastic%20astrocytoma) | III |
| T16 | Female | 64 | Right temporal lobe | [Anaplastic astrocytoma](https://dict.cn/Anaplastic%20astrocytoma) | III |
| T18 | Female | 65 | Right temporal lobe | Astrocytoma | III |
| T20 | Male | 47 | cerebellum | [Anaplastic astrocytoma](https://dict.cn/Anaplastic%20astrocytoma) | III |
| T27 | Female | 34 | Right temporal lobe | Astrocytoma | II |

**Table S6.** qRT-PCR primer sequences used in this study for the indicated genes

| **Genebank (ID)** | **Genes** |  | **Forward primer (5’-3’)** | **Reverse primer (5’-3’)** | **Product length (bp)** |
| --- | --- | --- | --- | --- | --- |
| NM_[020771](http://www.ncbi.nlm.nih.gov/entrez/query.fcgi?cmd=Search&db=Nucleotide&term=NM_001792).3 | *HACE1* |  | CTGCCAGAACGGTCACAAG | GACCATGACTGCAAGCAAAG | 113 |
| NM_[006164.4.3](http://www.ncbi.nlm.nih.gov/nuccore/NM_005228.3) | *NRF2* |  | ACACGGTCCACAGCTCATC | TCTTGCCTCCAAAGTATGTCAA | 99 |
| NM_005985.3 | *La/SSB* |  | GGGCCGGAACCTTAAAGATA | TTAGAAACTTGTCCCGTGGC | 135 |
| NM_[002133.3](https://www.ncbi.nlm.nih.gov/nuccore/NM_002133.3) | *HMOX1* |  | CTGCTCAACATCCAGCTCTTTG | ATCTTGCACTTTGTTGCTGGC | 117 |
| [NM_001025434.2](https://www.ncbi.nlm.nih.gov/entrez/viewer.fcgi?db=nucleotide&id=1676355479) | *NOQ1* |  | GGCAGAAGAGCACTGATCGTA | CACCACCTCCCATCCTTTCTT | 108 |
| NR_003142.4 | *18S* |  | CGCCGCTAGAGGTGAAATTC | CTTTCGCTCTGGTCCGTCTT | 52 |

**Table S7**. miRNA-specific RT primer sequences

| **Genes** | **RT Sequences（5′- 3′）** |
| --- | --- |
| *let-7b-3p* | GTC GTATCCAGTGCGTGTCGTGGAGTCGGCAATTGCACTGGATACGACgggaagg |
| *miR-129-5p* | GTCGTATCCAGTGCGTGTCGTGGAGTCGGCAATTGCACTGGATACGACgcaagcc |
| *miR-342-3p* | GTCGTATCCAGTGCGTGTCGTGGAGTCGGCAATTGCACTGGATACGACacgggtg |
| *miR-15b-3p* | GTCGTATCCAGTGCGTGTCGTGGAGTCGGCAATTGCACTGGATACGACtagagca |
| *miR-452-3p* | GTCGTATCCAGTGCGTGTCGTGGAGTCGGCAATTGCACTGGATACGACcacttac |
| *miR-548an* | GTCGTATCCAGTGCGTGTCGTGGAGTCGGCAATTGCACTGGATACGACcaaaaac |
| *miR-374c-5p* | GTCGTATCCAGTGCGTGTCGTGGAGTCGGCAATTGCACTGGATACGACagcactt |
| *miR-573* | GTCGTATCCAGTGCGTGTCGTGGAGTCGGCAATTGCACTGGATACGACctgatca |
| *let-7b-5p* | GTCGTATCCAGTGCGTGTCGTGGAGTCGGCAATTGCACTGGATACGACaaccaca |

**Table S8**. qRT-PCR primer sequences used in this study for the indicated miRNAs

| **miRNAs** | **Sequences（5′- 3′）** |
| --- | --- |
| Loop-F forward | ATCCAGTGCGTGTCGTG |
| let-7b-3p reverse | TGCTCTATACAACCTACTG |
| miR-129-5p reverse | TGCTCTTTTTGCGGTCTG |
| miR-342-3p reverse | TGCTTCTCACACAGAAATCG |
| miR-15b-3p reverse | TGCTCGAATCATTATTTGC |
| miR-452-3p reverse | TGCTCTCATCTGCAAAGAA |
| miR-548an reverse | TGCTAAAAGGCATTGTG |
| miR-374c-5p reverse | TGCTATAATACAACCTGCT |
| miR-573 reverse | TGCTCTGAAGTGATGTGTAAC |
| let-7b-5p reverse | TGCTTGAGGTAGTAGGTTG |
| U6 forward | GCTTCGGCAGCACATATACTAAAAT |
| U6 reverse | CGCTTCACGAATTTGCGTGTCAT |

**Table S9.** The sequences of siRNAs used in this study

| **siRNAs** | **Sense (5’-3’)** | **Antisense (5’-3’)** |
| --- | --- | --- |
| si-HACE1-854 | CCAAAUGUAUCAGGAGCAATT | UUGCUCCUGAUACAUUUGGTT |
| si-HACE1-382 | GCCAUGGUUAUGGCUGAUTT | AUCAGCCUAACCAUUGGCTT |
| si-NRF2-2113 | GCACCUUAUAUCUCGAAGUTT | ACUUCGAGAUAUAAGGUGCTT |
| si-NRF2-821 | CCCGUUUGUAGAUGACAAUTT | AUUGUCAUCUACAAACGGGTT |
| si-NRF2-1385 | GCCCAUUGAUGUUUCUGAUTT | AUCAGAAAACAUCAAUGGGCT |
| si-La/SSB-1136 | CAACAAGAAUCCCUAAACATT | UGUUUAGGGAUUCUUGUUGTT |
| si-La/SSB-775 | GGAAGCUAAAUUAAGAGCUTT | AGCUCUUAAUUUAGCUUCCTT |
| si-KEAP1 | GCGCCAAUGUUGACACGGATT | UCCGUGUCAACAUUGGCGCTT |
| si-NC | UUCUCCGAACGUGUCACGUTT | ACGUGACACGUUCGGAGAATT |

**Table S10.** miRNA mimics and negative controls used in this study

| mimics | **Sense (5’-3’)** | **Antisense (5’-3’)** |
| --- | --- | --- |
| hsa-miR-15b-3p | CGAAUCAUUAUUUGCUGCUCUA | TAGAGCAGCAAAUAAUGAUUCG |
| hsa-miR-129-5p | CUUUUUGCGGUCUGGGCUUGC | GCAAGCCCAGACCGCAAAAAG |
| micrONTM mimic Negative Control #22 ( miR01101) | | |

**Table S11.** The primers used in this study for plasmid construction

| **Constructs** | **Forward primer (5’-3’)** | **Reverse primer (5’-3’)** | **Restriction sites** |
| --- | --- | --- | --- |
| pcDNA(-)-HACE1/ HACE1 C876S | AATTTGCTAGCGCCACC ATGGAGAGAGCGATGGAGCAA | ATATAGGTACC TGCCATTGTGTAACCATAGCTG | *NheⅠ*& *KpnⅠ* |
| pcDNA(-)-HACE1△HECT | AATTTGCTAGCGCCACC  ATGGAGAGAGCGATGGAG | ATATAGGTACC  TGCTTTTGACACAACTTCACA | *NheⅠ*& *KpnⅠ* |
| pcDNA(-)-HACE1△ANK | AATTTGCTAGCGCCACC  ATGACACAGAATGAAGACCTC | ATATAGGTACC  TGCCATTGTGTAACCATAGCT | *NheⅠ*& *KpnⅠ* |
| La/SSB | AATTTGCTAGCGCCACC ATGGCTGAAAATGGTGATAATG | GCGGGATCC  TACTGGTCTCCAGCACCAT | *NheⅠ*&  *BamHⅠ* |

**Table S12.** The antibodies used in this study

| **Antibodies** | **Catalog#** | **Source** |
| --- | --- | --- |
| anti-IgG | ab6715 | Abcam |
| anti-HACE1 | ab32567 | Abcam |
| anti-Ubiquitin | ab33893 | Abcam |
| anti-GAPDH | M20006 | Abmart |
| anti-Histone H3 | ab1791 | Abcam |
| anti-Ki67 | 550609 | BD Pharmingen |
| anti-NRF2 | sc-722 | Santa Cruz |
| anti-La/SSB | sc-166274 | Santa Cruz |
| anti-KEAP1 | sc-33569 | Santa Cruz |
| anti-Tubulin | sc-73242 | Santa Cruz |
| anti-Calnexin | sc-46669 | Santa Cruz |
| anti-Myc tag | 05-724 | MILIPORE |

**Table S13.** The primers used in this study for luciferase reporter plasmid construction

| **Constructs** | **Forward primer (5’-3’)** | **Reverse primer (5’-3’)** | **Restriction sites** |
| --- | --- | --- | --- |
| pcDNA3-NRF2 IRES | AAATCAGGGAGGCGCAGCTC | GATGAGCTGTGGACCGTGTG | *KpnⅠ*& *BamHⅠ* |
| pmirGLO*-La/SSB* 3’-UTR | ATAGGTTTTAAACGACTTTT | TTTTTTTGCTCTTTTCATAT | *NheⅠ*& *XbaⅠ* |
